# Cybersecurity Attacks and Detection Methods in Web 3.0 Technology: A Review

**DOI:** 10.3390/s25020342

**Published:** 2025-01-09

**Authors:** Bandar Alotaibi

**Affiliations:** Department of Information Technology, University of Tabuk, Tabuk 47731, Saudi Arabia; b-alotaibi@ut.edu.sa

**Keywords:** Web 3.0, cybersecurity, metaverse, blockchain, decentralization, cryptocurrency, decentralized finance

## Abstract

Web 3.0 marks the beginning of a new era for the internet, characterized by distributed technology that prioritizes data ownership and value expression. Web 3.0 aims to empower users by providing them with ownership and control of their data and digital assets rather than leaving them in the hands of large corporations. Web 3.0 relies on decentralization, which uses blockchain technology to ensure secure user communication. However, Web 3.0 still faces many security challenges that might affect its deployment and expose users’ data and digital assets to cybercriminals. This survey investigates the current evolution of Web 3.0, outlining its background, foundation, and application. This review presents an overview of cybersecurity risks that face a mature Web 3.0 application domain (i.e., decentralized finance (DeFi)) and classifies them into seven categories. Moreover, state-of-the-art methods for addressing these threats are investigated and categorized based on the associated security risks. Insights into the potential future directions of Web 3.0 security are also provided.

## 1. Introduction

The concept of Web 3.0 has arisen recently as a response to the challenges posed by the current internet paradigm. These challenges include, but are not limited to, the monopolization of platform resources and violations of personal privacy. Web 3.0 is a new version of the internet that is being rebuilt via decentralized solutions such as blockchain and cryptography; its ultimate goal is to address current internet data ownership and value expression issues by employing distributed technology [1]. Gavin Wood (the cofounder of Ethereum) introduced the concept of Web 3.0 [2], which eliminates the need for centralized organizations, as seen in Web 1.0 and Web 2.0 [3]. Web 3.0 uses blockchain technology to establish a decentralized environment and to move data away from centralized organizations, enabling the construction of services and applications. The development of applications on a single server for processing business logic or storing user data in a database is unnecessary for Web 3.0 developers. Web 3.0 applications are hosted on various peer-to-peer (P2P) networks and distributed on decentralized networks created with blockchain technology.

In addition to the data ownership benefit noted above, the conversion of the internet from central settings to decentralized environments also has the following advantages:

- Open: Public communities store the data, which sustains the open network concept of Web 3.0. Additionally, Web 3.0 applications enforce a global view in which data are permanently visible to all participants.
- Trustless: Relying on a trusted third party is unnecessary when exchanging assets and data with unfamiliar users.
- Permissionless: Users cannot tie their identities to any specific platform. Additionally, authorization from the governing authority is unnecessary, giving users more freedom in their activities.
- Anonymous: Users can achieve partial anonymity by using different pseudonyms or storing data via blockchain.
- High availability: Web 3.0 delivers a high-availability environment, which decreases the possibility of server crashes and eliminates the single point of failure issue.
- Compatibility: Web 3.0 applications and services apply to a specific blockchain technology, such as Ethereum, and are compatible with all competitive blockchain technologies, such as Avalanche, Binance Smart Chain, Solana, and Oasis Network. Therefore, Web 3.0 can be integrated with existing blockchain technologies and emerging ecosystems.

Web 3.0 applications have recently become more common; more than 14,470 applications have been deployed on several platforms, and there are two million active users as of August 2023 [4]. Web 3.0 provides a comprehensive and user-friendly experience suitable for new users. Internet users only need to connect their wallets to their targeted sites to enjoy the Web 3.0 experience. These websites offer various activities for users to enjoy, including games, trades (e.g., NFTs [5]), and asset exchanges (e.g., DEXes [6]).

**Figure 1:**
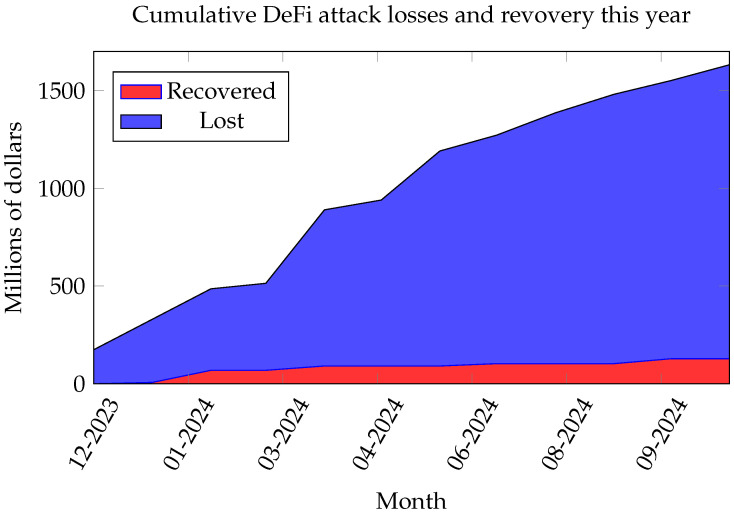
Cumulative DeFi losses and recovery from December 2023 to November 2024.

Despite the many advantages of Web 3.0, its decentralized nature and lack of centralized control introduce new risks and vulnerabilities. As Web 3.0 evolves and matures, its applications and services become increasingly susceptible to cybercrimes. In the last year, as shown in Figure 1, approximately USD 1.5 billion was stolen due to vulnerabilities and cyberattacks. According to De.Fi (i.e., a crypto antivirus and DeFi portfolio tracker tool) [7], the total DeFi funds lost in December 2023 was USD 174 million, and no fund was recovered. By June 2024, the losses had increased to USD 1.1 billion, and only USD 91 million was recovered. By November 2024, the cumulative losses reached USD 1.504 billion, while recovered funds were USD 128 million. This figure highlights the rapid financial risks related to DeFi ecosystems, illustrating the urgency for robust detection and prevention mechanisms. The appealing characteristics of Web 3.0—decentralization, anonymity, and lack of trusted third parties—can also be exploited by malicious actors for illegal activities. Web 3.0’s lack of technology standards and regulations creates space for cybercriminals to launch illegal activities such as wash trading, malicious attacks, fraud, and spam. This survey examines the cybersecurity landscape of Web 3.0, specifically focusing on decentralized applications. In contrast to the centralized nature of Web 2.0, Web 3.0 utilizes blockchain technology for decentralized, trustless, and permissionless interactions. The survey explores vulnerabilities and attack vectors in Web 3.0 applications, focusing on DeFi while excluding centralized systems. By focusing on the unique features of Web 3.0, this review seeks to clarify the security challenges present in decentralized environments and reviews solutions that might address these challenges. Although this survey focuses on DeFi as an exemplary domain for investigating Web 3.0 cybersecurity, the security issues and mitigation techniques analyzed are broadly relevant to other application areas, such as metaverse and health, which might encounter identical vulnerabilities, such as smart contract exploits and Oracle manipulation. We chose DeFi because it is mature and has well-documented security issues, which is an applicable application domain and can serve as a foundational representation for exploring Web 3.0 issues and mitigation approaches. This survey focuses on machine learning-based techniques for cybercrime and cyberattack detection in Web 3.0 because they are adaptable, scalable, and efficient at dealing with complex transaction patterns. Motivated by the evolution of Web 3.0 technology and its potential cybersecurity challenges, this survey makes the following contributions:

- A new categorization for cyberattacks in Web 3.0, focusing on decentralized finance (DeFi).
- A comprehensive overview of advanced detection methods from the past five years, focusing on machine learning techniques.
- A forward-looking discussion on key research challenges, including smart contract standardization, application-specific issues, AI-based detection methods, and underlying blockchain infrastructure issues.

The rest of this paper is structured as follows: Section 2 introduces the research methodology. Section 3 presents the history of the World Wide Web. Section 4 briefly explains the underlying Web 3.0 architecture. Section 5 specifies and classifies cyberattacks that target the mature Web 3.0 application, i.e., decentralized finance (DeFi). Section 6 analyzes the latest solutions to Web 3.0 security threats. Section 7 highlights shortcomings in current solutions and determines research gaps. Section 8 concludes this survey paper.

## 2. Research Methodology

A thorough search was performed across several reputable publishers, including IEEE, MDPI, Springer, and Elsevier. The search utilized a combination of keywords such as Web 3.0, blockchain, DeFi, cybersecurity, smart contracts, cyberattacks, cryptoassets, NFTs, and decentralization. The focus was on literature published mainly in the last five years to ensure the relevance and recency of the findings.

The criteria for including studies were as follows:

- The study should be published in peer-reviewed journals, at conferences, or in reputable academic sources.
- The focus should be on Web 3.0 technologies, blockchain, decentralized finance, or relevant cybersecurity risks.
- The focus should be on studies published within the last five years to ensure relevance to current Web 3.0 applications and associated security risks.

The criteria for excluding studies were as follows:

- This review excludes articles that are not directly related to Web 3.0 or cybersecurity.
- This review excludes articles that do not make theoretical or empirical contributions, such as those that lack sufficient analysis or consist of mere opinion pieces.

The screening process was conducted as follows. We systematically reviewed the articles’ titles and abstracts to firmly establish their relevance to the scope of this survey. Studies that successfully passed the title and abstract screening were analyzed in full to evaluate their content, methodology, and findings thoroughly. We extracted essential data on cybersecurity risks, methods, and proposed solutions from each study to effectively categorize and analyze the existing literature on Web 3.0 cybersecurity. The final review included studies that provided valuable insights into Web 3.0 security, focusing on their contributions to highlighting cybersecurity risks and potential countermeasures.

## 3. Background

**Figure 2:**
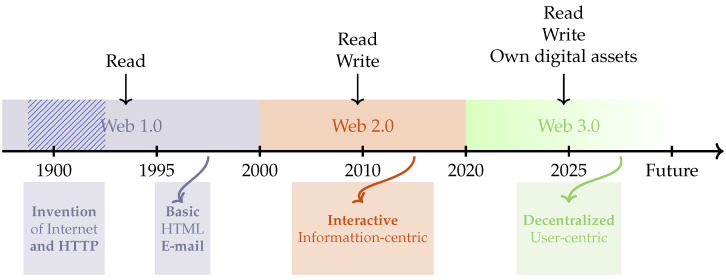
The evolution of the three Web eras and their essential characteristics.

This section presents the history of the World Wide Web. The Web was invented by Berners-Lee in 1989 and has undergone three eras, as shown in Figure 2: Web 1.0, Web 2.0, and Web 3.0. This section briefly summarizes the development processes and characteristics of these three eras. Table 1 shows the differences between the last two generations (i.e., Web 2.0 and Web 3.0).

### 3.1. The Evolution of the First Generation

Tim Berners-Lee invented the World Wide Web in 1989 at the European Organization for Nuclear Research (a.k.a. CERN) in Switzerland, introducing the initial proposition that started the internet era as an application. Berners-Lee and his research group presented the first website the following year: http://info.cern.ch/ [19]. Then, the backbone of the web page was invented (i.e., hypertext markup language (HTML) [20], hypertext transfer protocol (HTTP) [21], and Browser). Web 1.0 enables internet application users to access web server content and search for information. Web 1.0 users cannot edit content. The server is an integral part of the success of the Web 1.0 paradigm (it is the content provider).

### 3.2. The Evolution of the Second Generation

Web 2.0 is the current state of the internet, enabling users to generate and edit content. In the last twenty years, companies such as Facebook, Twitter, Google, and Amazon have used Web 2.0 to authorize users to post, comment, like, and upload content on these networks. Web 2.0 enables users to utilize the World Wide Web to interact with other users worldwide. The server is an intermediate between users, enabling users to store, transfer, and manage their data. However, in this generation, users’ data are stored at these social media/e-commerce companies, which encounter serious privacy issues.

### 3.3. The Conception of the Third Generation

The concept of Web 3.0 was introduced to overcome the disadvantages of Web 2.0. Berners-Lee et al. [22] propose a prototype applicable for Web 3.0 known as Solid to address the privacy leakage issue and the issue of large company monopolization. Solid is a decentralization platform introduced for social media applications and used to ensure the data independence of the social media application that manages the data access. In such settings, users store data in pods, and the application should comply with specific access protocols. Additionally, authentication and access control mechanisms are distributed to ensure authenticity and data privacy.

Users can communicate through different platforms and determine access rights to their data, which can be determined through the services they access. The Web 3.0 concept was subsequently standardized by Gavin Wood, who defined it as a system that integrates the World Wide Web with distributed networks, such as blockchain technology and smart contracts. In the research community, the most obvious characteristic of Web 3.0 is decentralization. In summary, Web 3.0 can be defined as a new era of the internet that is structured in a decentralized fashion, ensures value expression, and guarantees users’ data ownership. Web 3.0 employs the following principle: users must own and manage data and digital assets instead of large companies. Web 3.0 provides the following benefits: privacy preservation, decentralization, AI-empowered, distribution, and blockchain-based.

## 4. Web 3.0 Architecture

Owing to the lack of standardization for Web 3.0, there is no consensus among the research community and industries on the layers that Web 3.0 might constitute. Web 3.0 architecture will likely resemble its predecessors, using TCP/IP as its layering standard. This standard consists of five well-known layers: the Physical layer, the Data-Link layer, the Network layer, the Transport layer, and the Application layer. The main distinction is that Web 3.0 will implement different protocols at each layer than those used in Web 1.0 and Web 2.0. This research is not affected by layering issues because it focuses mostly on the security of the application layer, which must be included in any layering standardization that might be presented in the future. Many researchers propose different Web 3.0 architectures, including that proposed in [1]. This section briefly presents the most reasonable Web 3.0 architecture proposed in [1] to clarify what the Web 3.0 architecture might look like. The architecture consists of four layers: an infrastructure layer, an interface layer, a management layer, and an application layer.

### 4.1. Infrastructure Layer

This is the first layer of the Web 3.0 architecture that is responsible for data collection, storing, transmitting, and processing. The data sources would be greatly expanded in Web 3.0 (which emphasizes governance and decentralization [23]) to include the use of terminal devices from the IoT and feedback provided in real time by processors representing internet users. The transmitted data are analyzed at the network edge by specific nodes and might be stored via on-chain or hybrid on-chain and off-chain techniques to guarantee data transparency. In such settings, artificial intelligence (AI) is employed to increase storage computing efficiency and system security, protect privacy, and optimize strategies.

### 4.2. Interface Layer

The second layer of the Web 3.0 architecture aims to connect the physical and digital worlds. After collecting data from the infrastructure layer, this layer transforms the data into valuable digital assets. It also designates a value circulation system to encourage user involvement and optimize data utilization. The data value is specified by supply and demand, enabling users to take advantage of their contributions. Unlike the Web 2.0 paradigm, in which centralized organizations handle and coordinate private data, Web 3.0 enables users to have full control of their data. This feature is provided by decentralized identities capable of protecting privacy and ensuring appropriate access control. Additionally, emerging technologies can enable identity system decentralization [24] and smart data markets [25], as well as preserving the user’s privacy [26,27].

### 4.3. Management Layer

This layer aims to govern the lower layers (i.e., infrastructure and interface layers), implement incentives to encourage user participation [28,29], and track content generated by users. The management layer can also delete or correct erroneous or dated network traffic information that users store. Additionally, this layer is responsible for detecting and preventing malicious traffic from targeting the system. Moreover, AI can detect malicious network traffic and abnormal trading behavior and identify false or inappropriate information.

### 4.4. Application Layer

This layer might include several use cases from the metaverse [30], health care [31], and finance [32]. For example, as a use case in smart city environments, this layer might contain navigation apps that measure traffic conditions in real time and adjust routes on that basis. These applications must be secured to protect users’ privacy, systems, and network resources. AI and blockchain technology can enhance the user experience, improve privacy preservation, and increase efficiency. This study focuses on cyberattacks that target Web 3.0 applications and state-of-the-art solutions that detect or prevent these attacks.

### 4.5. The Interconnection Between Web 3.0 Layers in Addressing Cybersecurity Challenges

The four layers of Web 3.0 architecture can be closely integrated to create a secure and resilient environment that addresses the cybersecurity challenges inherent in a decentralized system. The infrastructure layer secures data collection, transmission, and storage, leveraging blockchain technology to ensure transparency and integrity. The interface layer then allows users to interact with these data, ensuring privacy and control over personal information through decentralized identities and access management. The management layer monitors the entire system, identifying and preventing malicious activities across the infrastructure and interface layers utilizing AI-based intrusion detection systems (IDSs)/intrusion prevention systems (IPSs) and other monitoring tools. Lastly, the application layer, which facilitates user interactions, integrates these security measures to protect user data, ensure transaction integrity, and prevent cyberattacks. These layers form a comprehensive framework that addresses the technological and governance challenges of Web 3.0, reducing risks such as malicious attacks.

### 4.6. Web 3.0 Cybersecurity Characteristics and Challenges

Web 3.0 is built on blockchain and distributed ledger technology (DLT), enabling trust and security through decentralized systems, cryptography, and consensus protocols like proof of work (PoW) and proof of stake (PoS). These protocols are adapted to provide secure blockchain networks against unauthorized modifications [33]. While these technologies strengthen cybersecurity principles like availability via distributed storage across peer-to-peer (P2P) paradigm, data integrity through transaction validation and cryptographic hashing, and non-repudiation through digital signatures [34], they also present challenges related to smart contracts’ vulnerabilities and Oracle manipulation issues. For instance, in DeFi applications, secure smart contracts are crucial to prevent exploits like reentrancy attacks and flash loan manipulations. DLT eliminates central authority reliance, supporting the “trustless” vision of Web 3.0 by distributing transaction validation across multiple nodes [35] while increasing vulnerabilities in decentralized systems, such as manipulating oracle-based systems. These characteristics enhance traditional cybersecurity models, like the confidentiality, integrity, and availability (CIA) triad and zero trust architecture (ZTA), while utilizing game theory to promote secure network behavior. Web 3.0 addresses traditional security concerns and requires new methods to address risks unique to decentralized environments.

## 5. Web 3.0 Cyberattacks

**Figure 3:**
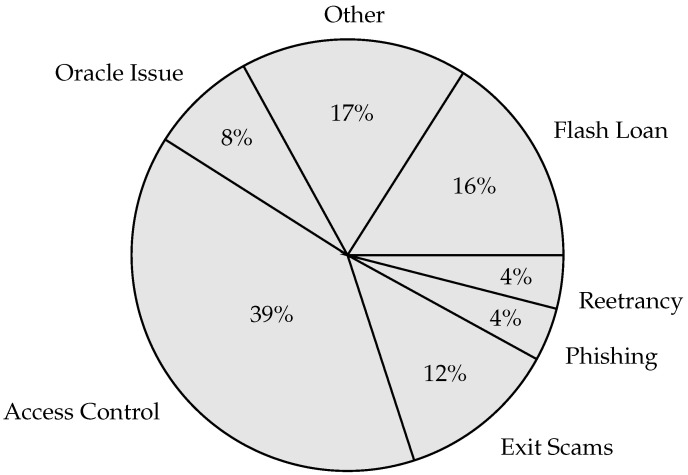
The main cyberattacks that targeted the Web 3.0 paradigm.

Web 3.0 encounters several cybersecurity issues that can be divided into cyberattacks and financial cybercrimes. Cyberattacks comprise technical vulnerabilities like Oracle manipulation, code exploits, and network-specific threats (e.g., Sybil and DDoS). These cyberattacks frequently expose financial applications to serious risks, leading to financial cybercrimes such as money laundering, scams, and wash trading. This section identifies the cyberattacks that target the main Web 3.0 application domain, particularly financial applications, and explores their connection to financial cybercrimes. Figure 3 shows the main cyberattacks that targeted the Web 3.0 paradigm last year (i.e., 2023) [36]. The figure highlights the widespread prevalence of attacks such as access control issues and flash loans, which should be associated with prioritizing risk mitigation approaches in Web 3.0 applications.

Financial crimes are offenses against someone’s property in which the criminal unlawfully transfers the victim’s money or any other kind of property to him- or herself or another person. Financial crimes include scams, money laundering, wash trading, and code exploits. These crimes affect victims because the victims lose their assets, and these crimes also pose a threat to the whole economic environment. Owing to the recent development of decentralized finance (DeFi), financial crimes have been diversified and complicated. As blockchain technology is deployed to provide other security benefits, many cybercriminals have found new vulnerabilities that enable them to launch various attacks, such as scams, identity theft, and money laundering [37].

The lack of adequate Web 3.0 standardization might lead to a hotbed of cybercrimes, facilitating the formation of financial crimes, including code exploits, scams, money laundering, wash trading, and unlawful applications and services. This subsection provides an overview of financial cybercrimes that have appeared since the emergence of applications that rely on decentralized solutions (i.e., those that could be part of Web 3.0 in the future).

### 5.1. Scams

The concept of a scam was ubiquitous before the internet was invented. A scam can be defined as a dishonest means used by fraudsters to obtain something that has value from others or a confidence trick performed by a criminal group, person, or organization. Before the emergence of the internet, scams transpired regularly in offline social interactions. In recent decades, scammers have exploited the internet through email and social networks to initiate their hustle. Recently, with the appearance of the cryptocurrency market, which enables anonymity, scammers have committed untraceable cryptoasset scams and tried to swindle investors for unlawful gains. Scams on Web 3.0 can be classified into phishing attacks, Ponzi schemes, fake exchanges, giveaway scams, and rug pulls [38].

**Figure 4:**
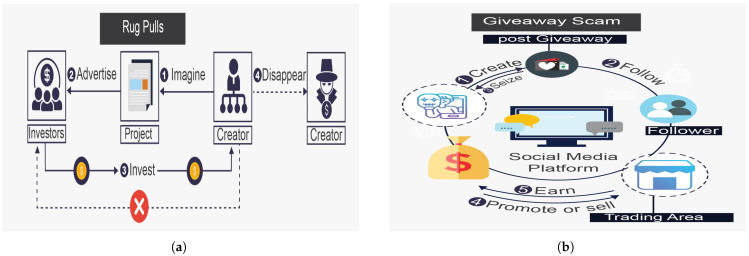
The most common scam in the DeFi ecosystem, rug pull (**a**), and an emerging scam, giveaway scam (**b**).

**Figure 5:**
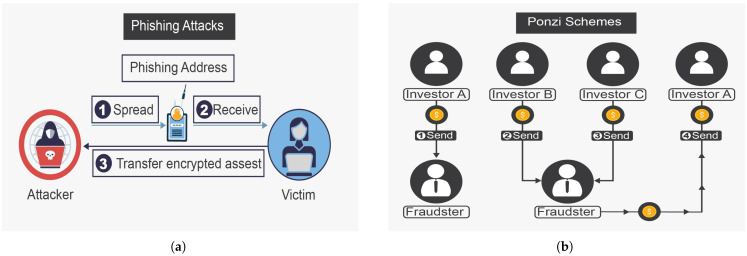
Phishing (**a**) and Ponzi schemes (**b**) are two devastating scam attacks in the DeFi environments.

- The rug pull, shown in Figure 4a, is a devastating attack that depletes investments from decentralized exchange (DEX) liquidity pools or results from the sudden abandonment of a project without warning signs [39]. This attack usually occurs in DEX and is considered a typical DeFi scam. Scammers deceive investors by investing heavily in liquidity tools and creating and posting attractive advertisements on social media. Once investors deposit tokens in these liquidity pools, the tokens are withdrawn from the pools. A well-known rug pull attack manipulates the token price to match the attacker’s reserves; this is performed by artificially decreasing the token’s value, often causing it to plummet to zero. In 2021, rug pull attacks in the DeFi ecosystem resulted in approximately USD 2.8 billion in losses, making it the largest scam in DeFi, equivalent to 37% of all scam revenue that year [40].
- The giveaway scam, shown in Figure 4b, is a new scam that functions by constructing websites that seem legitimate to investors or breaking into accounts on social media that have large numbers of followers to publish free giveaway plans, luring investors into thinking that any investor who wires a given amount of funds will be paid back double or more that amount. More specifically, the attacker creates a legitimate-looking website, announces the free giveaway on the website, and uses as many distribution channels (such as online social networks (OSNs), including Facebook, Twitter, and YouTube) as possible to circulate the website URL [41].
- Phishing attacks are among the most important and influential social engineering attacks in the DeFi ecosystem. As shown in Figure 5a, phishing involves an attacker attempting to lure the victim and gain access to private data after earning his or her trust. The attacker can include bogus personal information in the deceiving venue to deceive the investor into thinking that it is legitimate. The victim perceives this behavior as legal, leading them to trust the attacker and engage in cryptocurrency investment and exchange [42].
- Ponzi schemes introduce greater risk than other online cybersecurity attacks do. As shown in Figure 5b, the owner acquires the first Ethereum token, in which the attacker lures the investors to believe that they will gain high return rates with minimal risk, while the attacker is performing fraudulent investment behavior (i.e., Ponzi scheme). This scheme is a deceitful procedure utilized by attackers to collect digital assets from later investors to pay back earlier investors. Thus, for this attack to succeed, new investors must generate money to compensate for old investors. Once new investors stop coming in and there is not enough money to go around, Ponzi schemes ultimately collapse. Ponzi schemes involve unethical individuals defrauding unsuspecting victims with promises of high profits in exchange for their investment [43].

Web 3.0 is decentralized in nature, allowing scammers to target users directly and without oversight from traditional banks or financial institutions. In traditional systems, intermediaries typically have fraud detection and prevention techniques in place. However, in Web 3.0, the absence of these intermediaries allows attackers to more easily execute phishing attacks, giveaway scams, rug pulls, and Ponzi schemes without being detected or stopped immediately.

### 5.2. Code Exploit

In recent years, blockchain technology has been utilized in many fields and services, including the financial sector, health care, and metaverse, due to the success of Bitcoin. These services and applications utilize blockchain technology to transfer and store valuable information, so they have become targets for cybercriminals [44]. Thus, attackers exploit the vulnerabilities of the blockchain system to launch their attacks. The insufficient protection of private keys by blockchain account owners or in cryptocurrency exchanges is one of the main vulnerabilities that attackers can exploit to steal digital assets. Blockchain systems are also susceptible to other vulnerabilities related to blockchain protocols and smart contract implementations [45,46]. Protocol configuration weaknesses become a serious security issue when blockchain designers underestimate the full implications of features constructed into their technology. Additionally, digital assets (e.g., cryptocurrency) can be stolen by exploiting smart contract vulnerabilities (e.g., integer overflow or underflow).

Additionally, there are situations in which code is maliciously explored in the finance realm. In September 2020, an NFT game known as Eminence was created by the developer of Yearn Finance; this game included a token called EMN. Some investors exposed the token, and in a few hours, they minted USD 15 million worth of EMN. The investors used a smart contract to enable players to exchange stablecoin DAI for in-game purchases. Unfortunately, a hacker employed a flash loan to deplete the contract funds. This behavior dropped the token price dramatically. Even though NFTs use blockchain technology, marketplaces, and exchanges, including OpenSea and Rarible, they function in a centralized setting, preventing them from benefiting from peer review techniques that can determine and correct errors. Therefore, they are exposed to code exploit threats. In 2021, 42 NFTs vanished because of the weakness exploited by attackers in the OpenSea token, which cost USD 100,000 [47].

Another code exploit is the reentrancy attack, which occurs when an external call alters the smart contract workflow. Because of this fallback, the malicious external call can alter the order of the target’s execution. A malicious instance can recursively call back the same function if a contract alters the variable after an external call, but the variables are not updated [48]. For example, in 2016, the reentrancy vulnerability was exploited in smart contracts’ source code, enabling an attacker to obtain more than USD 60 million worth of Ethereum. In 2018, a similar instance occurred in the SpankChain contract, in which the reentrancy vulnerability was exploited, and USD 40,000 was unlawfully withdrawn. The same vulnerability coexists with several decentralized autonomous organization (DAO) projects, such as Uniswap and Lendf [49]. Thus, this security weakness may provoke uncorrectable project failures and investor losses. Smart contract applications are often undermined by reentrancy bugs, which can be exploited and undermine trust [50].

In Web 3.0, smart contracts automatically execute without human intervention, offering a significant advantage over traditional systems and enhancing transaction efficiency and trust [51]. Despite the automation and open-source nature of smart contract code, attackers can exploit these features. Vulnerabilities, such as reentrancy in smart contracts, arise from the decentralized and code-driven nature of Web 3.0 platforms. These vulnerabilities can be exploited without oversight, and the blockchain’s immutability makes it nearly impossible to reverse or alter transactions once an exploit occurs.

### 5.3. Oracle Manipulation

An oracle is black box software that presents a source of truth utilized by other systems. Traditionally, an oracle provides an anticipated result for a specific test case. Blockchain technology has highlighted the need for a source of truth to validate information outside of the calling contract, and oracles can provide that service. A smart contract can be used to bet on the U.S. presidential election, with an oracle ensuring that the election’s outcome settles payments (off-chain oracle). On-chain oracles use data available only on the blockchain to provide information.

In DeFI, traditional contractual agreements are replaced by smart contracts. Smart contracts provide two effective benefits over their traditional contractual agreement counterparts:

- Transparency: Smart contract codes are visible to the public on the blockchain. Therefore, investors can review the inner workings of their financial assets.
- Control: Investors in DeFi who interact with smart contracts can control their assets. This differs from traditional financial systems in which brokers, banks, and other financial organizations manage assets.

An oracle enables smart contacts to bridge the real world and Ethereum. Therefore, an oracle is essential for smart contract evolution in the DeFi paradigm [52]. A well-known use case of an oracle in DeFi enables smart contracts to obtain a price feed for a given asset. The asset price is utilized for lending, trading, or borrowing via smart contracts. Unfortunately, obtaining price information accurately, reliably, and concisely is difficult. The source of truth might be manipulated by adversaries utilizing vulnerabilities in the oracle system architecture on smart contracts and, eventually, obtaining assets. Indeed, the loss of assets in the blockchain is irreversible because of the immutable essence that blockchain provides.

Web 3.0 significantly depends on oracles to integrate external data into smart contracts, effectively linking off-chain information with blockchain transactions [53]. Oracles are essential for DeFi applications, which do not have centralized intermediaries that traditionally verify data, such as banks or financial institutions [54]. Decentralization promotes trustlessness but also allows adversaries to manipulate the information that oracles depend on. Oracle is designed to trust external data without human oversight, making it vulnerable to attacks. Attackers can exploit weaknesses in oracle systems or manipulate data inputs. The lack of centralized verification methods makes manipulation easier, as there are fewer checks and balances to identify and prevent malicious actions.

### 5.4. Wash Trading

The market manipulation behavior known as wash trading has emerged in conventional financial scenarios and is categorized as a crime worldwide [55]. Wash trading can be defined as actions that are performed repeatedly to trade assets to supply misleading information to other traders. These actions cause an expansion in fake trading volume and initiate a false sense of affluence.

Wash trading in finance appears in many forms and markets, such as cryptocurrencies, the NFT market, and the ERC20 token market. Inflating trading volume accusations have recently emerged because of suspicious exchanges. The third largest cryptocurrency exchange in South Korea, known as Coinbit, was blamed in August 2020 by police for fabricating more than 99% of its trading volume [56]. Additionally, wash trading in the NFT market is prevalent. For example, on one of the well-known decentralized NFT trading platforms known as LooksRare, 95% of the activities are associated with trading [37].

Wash trading in NFT emerges in two forms: the first occurs in the advance of a new NFT collection going public by falsifying trading volume, and the other is intended to gain additional token dividends through NFT transaction wash trading. One of the provisions employed by centralized NFT trading platforms such as OpenSea is to maintain at least 100 Ethereum transaction volumes, which is high for recently established collections. This provision might facilitate deceptive transactions in which attackers can artificially inflate transaction volumes by performing fictitious transactions between numerous accounts. It has been reported by Chainalysis that egregious double trading of three similar NFTs transferred between two wallets has occurred, trading approximately 650,000 Ethereum and commanding USD 114 million in transaction fees. The effect of this double trading is the loss of about USD 185.5 million worth of tokens from the NFT trading market. Thus, wash trading affects the valuation of digital assets and can manipulate the market. Wash trading is difficult to detect and prevent.

Wash trading, which creates misleading trading volume to deceive market participants, is worsened by the decentralized nature of Web 3.0 markets. In centralized exchanges, regulators and monitoring systems can identify suspicious trading patterns, but Web 3.0’s DEXs and NFT platforms often lack the oversight necessary to prevent wash trading [57]. The lack of intermediaries on these platforms means that there is no central authority to monitor or control market behavior. This absence can be exploited by attackers who conduct artificial trades between wallets they control, inflating transaction volumes, and misleading other investors. This lack of monitoring in decentralized systems allows traders to manipulate markets without being caught by regulators.

### 5.5. Money Laundering

**Figure 6:**
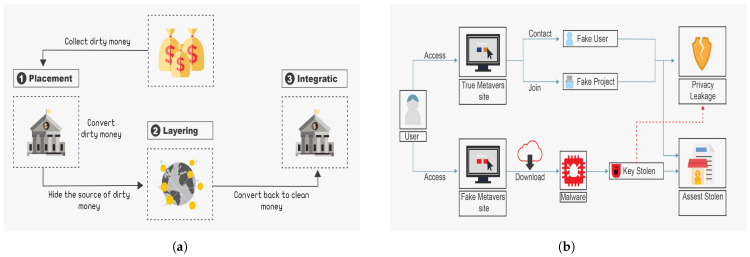
Two widely recognized forms of financial corruption that significantly impact the global economy: (**a**) hiding illicit funds using money laundering strategy and (**b**) cybercriminals misinterpreting digital assets using flash loan attacks.

Money laundering is a profound form of financial corruption that harms the global economy and feeds other crimes, such as drug trafficking and arms dealers. Money laundering is defined by the Association of Certified Anti-Money Laundering Specialists as obtaining the proceeds of crime and hiding their illegal origin to utilize them for lawful or unlawful activities [58]. Money laundering is the procedure used to make dirty money look clean, as shown in Figure 6a. This procedure can be categorized into three phases: placement, layering, and integration [59]. First, illegal funds are secretly directed into the lawful financial system. To hide the origin of the illicit funds, complex financial transactions are then employed by transferring money via multiple accounts to cause confusion. Finally, additional transactions are made to integrate the illegal funds into the financial system until the dirty money appears clean. Currently, many financial organizations enforce anti-money laundering (AML) policies to prevent harmful activities that have tremendous negative financial consequences.

In the cryptocurrency world, digital assets such as NFTs are widely deployed. However, lawful regulation of transactions is still in its infancy [60]. Money laundering potential is very high; with the emergence of land assets and wearables such as clothing and accessories designed to imitate avatars, attackers are most likely to utilize these assets for illicit fund processes. In 2021, sales of cryptocurrency land assets on blockchain-powered virtual reality platforms such as Cryptovoxels, Decentraland, Sandbox, and Somnium Space exceeded USD 500 million. For example, in 2021, the average land value in Decentraland was in excess of tens of thousands of dollars because Decentraland sold millions of dollars worth of plots, which indicates a potential means by which unlawful money can be wired. Additionally, unlike traditional purchases of land, purchasing DeFi land usually mandates just a cryptoasset address and enough funds (i.e., no need to retain a KYC check). This condition makes it easy for money launderers and criminals to launch attacks.

On the other hand, with wearable device assets, at the end of 2023, the market reached approximately USD 3 trillion. Offenders can purchase one wearable device from one metaverse and then move it to another, cashing out via secondary sales, creating untraceable money flow across multiple blockchains. These data indicate that as DeFi assets continue to grow, illegitimate users will most likely utilize them as a primary conduit for laundering assets that could be transferred from traditional activities or other crypto-based crimes. Additionally, offenders can disguise the origin of these assets by exchanging them for DeFi assets (e.g., land or wearable device assets) [37]. With the rise of cryptocurrencies, methods for detecting and preventing money laundering are needed.

The anonymity and decentralization of Web 3.0 create a favorable environment for money laundering. Traditional financial systems usually have KYC and AML protocols in place to monitor and verify transactions. In Web 3.0, the lack of intermediaries and the pseudonymous nature of transactions, such as those in DeFi, enable attackers to hide the origins of illicit funds, making it easier to launder them [61]. This process is subject to less scrutiny than that imposed by centralized financial institutions.

### 5.6. Flash Loan

A flash loan is a recent service released into the DeFi paradigm to enable users to apply for a no-collateral loan, as shown in Figure 6b. This service offers convenience to users; however, it allows attackers to initiate malicious attacks targeting a variety of digital assets [62]. An analysis of recent DeFi attacks revealed that approximately 50% of the current threats in the DeFi ecosystem involve flash loans [63]. Therefore, the flash loan attack is considered the most well-known DeFi attack. Thus, in this subsection, three well-known types of flash loan attacks (price manipulation [64], pump and arbitrage [65], and reentrancy [66]) are discussed.

- Price manipulation: This attack is one of the most well-known flash loan attacks. In this attack, cybercriminals misinterpret the documented price and transfer the misinterpretation via a malicious custom function, causing the targeted user to obtain inaccurate price information. In essence, the targeted user is misled about the digital asset’s high or low value. In general, a price manipulation attack involves the following steps: preparing a target digital asset, inflating this asset valuation, profiting from a targeted trader, and retaining the original asset price.
- Pump and Arbitrage: This attack generally involves making a profit by exploiting price differences on various exchanges for the same digital asset. Unlike real-world markets (i.e., those that react instantaneously to occurrences), the DeFi ecosystem does not react fast enough to occurrences on blockchain technology. Given such inefficient designs, cybercriminals can sell or buy digital assets at distinct prices, resulting in illegal financial gains. Cybercriminals can profit (i.e., obtain arbitrage) by using flash loans without owning any digital assets beforehand. Specifically, if the price difference is noticed, cybercriminals can quickly borrow numerous digital assets via a flash loan service and then engage in arbitrage.
- Reentrancy: This vulnerability is common in traditional smart contracts, leading to notorious decentralized autonomous organization (DAO) attacks [67]. This attack is launched as follows: A cybercriminal targets a victim’s smart contract and utilizes a given function to convey money to a malicious contract. The smart contract’s default settings force the malicious contract to be automatically revoked. The malicious contract can set a criminal procedure via the fallback function (i.e., revoking the victim’s smart contract one more time to execute an illicit second-time transfer). Because the current operation of the victim’s smart contract is waiting for the first-time transfer to terminate, the amount would not be withdrawn from the malicious contract balance yet, making the victim’s smart contract mistakenly consider that the malicious smart contract still has sufficient balance and thus to convey money to the victim’s smart contract one more time.

Flash loans offer convenience for legitimate users, but they can also be misused by attackers. DeFi’s decentralized nature and lack of intermediaries leave no central authority to prevent flash loan exploits or track malicious activities. Attackers can exploit flash loans to manipulate asset prices and engage in arbitrage across decentralized platforms [68]. Due to the trustless and decentralized nature of these transactions, victims, and platforms find it increasingly difficult to respond quickly or reverse the consequences of an attack.

### 5.7. Network Threats

- Distributed Denial of Services Attacks: Web 3.0 application domains consist of tiny wearable devices; attackers can compromise these devices and utilize them as part of a botnet, such as the Mirai botnet [69], to launch many DDoS attacks in a short period to overwhelm centralized servers and to make networks unresponsive and services unavailable. Additionally, owing to blockchain-constrained communication pressure and limited storage space, some NFT features could be employed on off-chain systems in practical services, which makes them vulnerable to adversaries who might launch DDoS attacks to prevent the availability of NFT systems. When establishing Web 3.0 services, the centralized model that relies on cloud computing is convenient for managing users or avatars and reduces operational costs. However, it can be susceptible to a single point of failure driven by DDoS attacks and physical root server damage [70]. Additionally, it presents transparency and trust issues related to the trustful exchange of virtual goods, digital assets, and virtual currencies through diverse virtual environments in Web 3.0.
- Sybil Attacks: Attackers can carry out attacks by stealing or pretending to be someone else, significantly impacting various Web 3.0 services such as blockchain consensus, reputation services, and voting-based services in digital governance. This can decrease the system’s effectiveness or even allow the attacker to take control of the entire Web 3.0 paradigm. Attackers can outvote legitimate nodes by launching sufficient Sybil identities to reject the transmission or receipt of some blocks, preventing other nodes in Web 3.0 from accessing the blockchain network.

The lack of a central authority makes it difficult to concentrate defenses, which complicates protection against DDoS attacks. Such attacks in decentralized environments degrade or deny the availability of services such as NFT platforms or blockchain nodes, where centralized protection mechanisms would typically mitigate these risks [71]. Web 3.0’s decentralized networks are based on the assumption that most participants are trustworthy. Without intermediaries, there are fewer ways to verify the legitimacy of individual nodes or participants. This facilitates attackers’ ability to flood the network with false identities, thereby manipulating voting systems or consensus processes. Attackers could control multiple nodes, skewing voting outcomes in decentralized governance systems or disrupting blockchain consensus. The absence of intermediaries for identity verification makes Web 3.0 networks vulnerable to Sybil attacks, which can severely compromise trust and functionality.

### 5.8. Compound Attacks

Web 3.0 environments are specifically vulnerable to compound attacks in which various attacks are integrated to expand their effect. For instance, an attacker manipulating an oracle could first feed false price data. Then, a flash loan attack can be performed, taking advantage of the manipulated price to gain financial benefit. Additionally, attackers could pair a reentrancy attack on a smart contract with phishing to target traders’ wallets and withdraw their funds. It is difficult to detect or prevent compound attacks since they propagate through multiple architecture layers, often taking advantage of the lack of interconnection between oracles, smart contracts, and decentralized applications. For example, launching a constructive DDoS attack on an oracle’s data feed can disrupt smart contracts dependent on that feed at the same time the attack is launched, which might eventually cause failure to the system.

## 6. Web 3.0 Cyberattack Detection and Mitigation

This section presents the state-of-the-art solutions proposed to detect or prevent cyberattacks in Web 3.0 (the DeFi application domain, in particular).

### 6.1. SCAM Detection

Compared with other types of cybercrime, cybersecurity threats introduced by Ponzi schemes pose a very high risk. These defrauding activities, such as Ponzi schemes, have grown rapidly, mostly in developing countries with high poverty rates. Numerous individuals have fallen prey to scammers, resulting in substantial financial losses. There have been many attempts to automatically identify Ponzi schemes via supervised and unsupervised machine learning techniques as shown in Table 2. However, these methods encounter many challenges, such as the reliance on transaction records, the limited number of collected samples, and unsatisfactory performance. To address these challenges and detect Ponzi schemes efficiently and accurately, Onu et al. [43] introduced an approach to detect Ethereum Ponzi schemes via machine learning. The authors compared three machine learning algorithms for that purpose, namely, the neural network, random forest, and k-nearest neighbor algorithms, and evaluated these algorithms via a dataset of 20,000 Ethereum transactions. The comparison results show that the RF model performed better than the other two models did, achieving a class score of 88.33%, an accuracy of 94%, and an overall score of 96.7%. The authors also presented a feature selection mechanism to reduce the number of features and improve the detection time. The feature selection mechanism reduced the number of features from 70 to 10, which improved the detection time and maintained high accuracy.

Vassallo et al. [72] proposed a malicious activities detection method targeting cryptocurrency infrastructures, including transaction and account levels. The malicious activities include Ponzi schemes and scams. The authors introduced a detection technique called adaptive stacked eXtreme gradient boosting (ASXGB), a customized version of eXtreme gradient boosting (XGBoost) for dynamic environments. The authors also compared their proposed approach with heuristic data sampling and tree-based ensemble methods. The proposed method outperformed the tree-based ensemble when tested on both transaction and account levels. Additionally, the synthetic minority oversampling (SMOTE) and neighborhood cleaning rule (NCL) data sampling techniques enhance the transaction level result in terms of recall.

Xiang et al. [73] constructed and published a recent dataset of Bitcoin transactions consisting of 13 types of Bitcoin addresses (classes), five indicator varieties, 148 attributes, and 544,462 labeled samples. The authors then compared several baseline machine learning classifiers, namely, random forest, decision tree, k-nearest neighbors, multilayer perceptron, and XGBoost, to validate the appropriateness of the dataset to detect multiple classes of suspicious activities in cryptocurrency platforms. The experimental results show the suitability of the dataset to detect multiclass cybersecurity threats targeting the Bitcoin network, which yielded reasonable accuracy rates ranging from approximately 93% to 97%. The authors also explored the extracted features and introduced a k-hop subgraph composition technique to generate a k-hop subgraph utilizing the whole Bitcoin transaction graph produced from the directed heterogeneous multigraph initiated from a given Bitcoin address (i.e., a general transaction linked to a criminal investigation). Additionally, the authors examined Bitcoin address behavior patterns via the extracted features.

Li et al. [74] introduced a temporal transaction aggregation graph network (TTAGN) to detect scams on Ethereum. The authors created an edge representation of the Ethereum transaction network by employing a temporal relationship of documented transaction records between nodes. The authors aggregated the edge representations around the node to combine topological interactive relationships (also known in the edge2node unit as trading features). The authors also merged trading features into statistical and structural features extracted from graph neural networks to distinguish scamming addresses from legitimate ones. The proposed method was evaluated on a real-world dataset and outperformed the state-of-the-art methods, achieving an AUC of 92.8% and an F1 score of 81.6%.

Fu et al. [75] proposed a new graph neural network-based account identification approach to detect scams in cryptocurrency platforms. The authors integrated an imbalanced data processing scheme for graphs to measure cryptocurrency transaction time. The proposed framework first obtains time features from the transaction graph via attention and LSTM procedures. The time features are combined with baseline features and fed into merged GCN and SMOTE models to classify phishing. The proposed method was evaluated on a customized dataset and showed promising results, achieving a classification accuracy of 97.22% and an AUC of 96.67%.

Elmougy et al. [76] introduced a holistic applied artificial intelligence approach to identify fraud in the Bitcoin network and presented an extended version called ELLiptic++ of the well-known cryptocurrency transactions dataset (i.e., the Elliptic). The new dataset comprises 822,000 Bitcoin wallet addresses associated with 56 features and 1,270,000 temporal interactions. The purpose of this dataset is to enable researchers to detect fraudulent transactions and illicit addresses in cryptocurrency platforms. The authors leveraged four categories of graph data to detect fraud in cryptocurrency platforms: the transaction-to-transaction graph that elucidates the money flow in the cryptocurrency network, the address–transaction graph that reveals the bidirectional money flow through transactions and addresses, the address-to-address interaction graph that shows the transaction flow types between cryptocurrency addresses, and the user entity graph that presents cryptocurrency addresses defining unique cryptocurrency users. The authors executed fraud detection schemes via the four graphs and machine learning techniques. The experimental results demonstrate the effectiveness of adding improved features (particularly address–transaction graphs and address-to-address graphs) in detecting illicit addresses and transactions, obtaining a detailed understanding of the main cause of money laundering vulnerabilities, and enabling researchers and cybersecurity experts to develop strategies for fraud detection and mitigation.

Wahrstatter et al. [77] investigated the whole Bitcoin user graph to recognize suspect accounts entangled in illicit actions. The authors presented new features that can precisely help determine suspicious activities. The authors employed an unsupervised machine learning method (i.e., a clustering algorithm) to analyze the network thoroughly. The proposed clustering algorithm was employed for a Bitcoin user graph, adjusted for CoinJoin, to investigate the network at a user-centric level and enhance privacy. The authors evaluated the proposed method on a customized dataset and reported that it detected most illegitimate activities with suspicious clusters. The researchers found that accounts linked to suspicious activities tend to have short paths to CoinJoin wallets and exhibit outlier behavior.

Garin and Gisin [78] compared supervised machine learning classifiers such as decision trees, random forests, gradient boosting, and logistic regression to identify suspicious activities in Bitcoin. The authors also balanced the classes in the dataset, selected the best possible hyperparameters, and implemented active learning to improve performance. The authors also applied a feature selection method to choose the most important features and reduce the detection time. The comparative results show that the gradient-boosting ensemble method outperformed the other methods in terms of various evaluation metrics.

Aziz et al. [79] proposed a deep learning-based approach that uses a unique metaheuristic optimization strategy to detect suspicious transactions in Ethereum. The framework combines deep learning with optimized genetic algorithm-cuckoo search (GA-CS). The framework uses the GA in the exploration step to address the deficiency issue in cuckoo search. The authors conducted a comprehensive experiment to evaluate the performance of their framework and compare it with several well-known supervised machine learning algorithms for detecting suspicious behavior on Ethereum: light gradient boosting machine (LGBM), support vector machine (SVM), random forest (RF), multilayer perceptron (MLP), extreme gradient boosting (XGBoost), k-nearest neighbors (KNN), and logistic regression (LR). The proposed framework and SVM achieved better results in terms of accuracy than the other methods did. The hybrid deep learning and optimization strategy achieved better accuracy than the random forest models (99.71% versus 98.33%).

Nayyer et al. [80] combined various machine learning classifiers in an ensemble method to detect suspicious Bitcoin activities. The ensemble method combines four famous classifiers: naive Bayes, KNN, decision tree, and random forest. The proposed framework uses a combination of adaptive synthetic and Tomek link balancing methods to balance the unbalanced dataset. The authors performed hyperparameter tuning via grid search, random search, and Bayesian optimization to enhance the classification results. The authors employed Shapley additive explanation (SHAP) to interpret the ensemble model predictions. The proposed framework was assessed according to seven evaluation metrics: accuracy, false positive rate (FPR), execution time, F1 score, area under the curve–receiver operating characteristic (AUC-ROC), recall, and precision. The proposed ensemble method yielded high results, achieving 96% precision, 98% recall, 97% F1 score, 97% accuracy, 99% AUC-ROC, and 3% FPR.

Pragasam et al. [81] introduced a technique to reveal the anonymity level in cryptocurrency platforms such as Ethereum by exploring multiclass machine learning algorithms to classify Ethereum’s externally owned accounts (EOAs); this can be accomplished via transaction pattern activity linked with account addresses. The authors utilized the Ethereum crypto dataset developed by Google BigQuery to analyze the classifiers. The authors constructed customized data from address profiles via address transactions in this dataset. The customized dataset contains 4371 samples, which have been used to predict address categories and evaluate various machine learning algorithms, such as gradient boosting, XGBoost, and random forest.

The best method was the XGBoost classifier, which achieved an accuracy of 75% and a macroaveraged F1 score of 69%. The second best-performing algorithm was random forest, which yielded an accuracy of 73.7% and a macroaveraged F1 score of 64%. The third best-performing algorithm was gradient boosting, which achieved an accuracy of 73% and a macroaveraged F1 score of 64%. The proposed model proved its effectiveness in predicting Ethereum wallet address categories such as scamming, phishing/hacking, exchange, and initial count offering (ICO) wallets through transaction behavior. However, the performance of the tested algorithms was not satisfactory because of the small number of samples that the authors used to validate these well-known machine learning algorithms.

Scam detection methods, especially those that try to detect phishing attacks and Ponzi schemes, are divided into approaches designed to focus on time efficiency and approaches that concentrate on high accuracy. Ensemble-based methods like the adaptive stacked XGBoost-based and neural network-based methods yield high precision while demanding significant computational resources; this demand makes similar approaches unpractical in real-world applications. Clustering-based approaches provide lightweight solutions to detect suspicious behavior but always produce high FPRs. The TTAGN is an innovative approach introduced to fill the gap between complexity and precision by integrating transaction patterns’ structural and temporal characteristics. Future work can focus on enhancing while paying attention to scalability by leveraging hybrid approaches that merge the clustering methods that are known to be time-efficient and graph-based techniques that are known to be highly precise.

### 6.2. Code Exploit Detection

In DeFi, lenders can supply flash loans to borrowers. This strategy is valid only within a blockchain transaction, and the borrower should repay the loan and transaction fees by the transaction’s due date. Flash loans differ from traditional loans because they do not require upfront collateral deposits. Hence, adversaries employ flash loans to collect immense assets after discovering and exploiting DeFi protocol vulnerabilities. This subsection analyzes the approaches that attempt to detect code exploits, and Table 3 compares these approaches. Chen et al. [82] presented an automated approximation technique to investigate malicious transactions that utilize flash loans to exploit the vulnerabilities of DeFi protocols. The authors introduced two numerical approaches (nearest-neighbor interpolation and polynomial linear regression) to approximate the operational behaviors of the DeFi protocol. The authors developed an optimization query to explore malicious behavior formed by a series of function invocations with optimal parameters on the basis of the approximated functions of the DeFi protocol that offer maximum profit. The authors presented a new counterexample-driven refinement method to enhance the approximation performance. They utilized a tool called FlashSyn to implement their framework. They validated their framework via 16 DeFi protocols that were vulnerable to flash loan attacks and two DeFi protocols used in the Damn Vulnerable DeFi platform. The authors employed FlashSyn to synthesize malicious behavior for 16 benchmarks. Then, they tested the identification performance of their framework on 16 successful cases in which the tool identified attack vectors that achieved more increased yields than those utilized by historical attackers in three cases. The tool also identified attack vectors in ten cases, showing its effectiveness in identifying potential flash loan invasions.

Wang et al. [83] proposed a deep learning-based framework to detect and classify Defi attacks, particularly flash loan and price manipulation attacks, that exploit DeFi applications’ logic vulnerabilities to gain profit. The proposed framework employs a neural network method that consists of a local model, a global mode, and an ensemble mechanism and is trained to classify DeFi attacks. The authors first normalized the unstructured emitted events; then, they enriched the transaction features of normalized emitted events via the global model and extracted the semantic features of the emitted events via the local model. Subsequently, they merged both groups of features via the ensemble model to detect DeFi attacks efficiently. The authors evaluated their framework via customized data from 50,910 DeFi transactions. The experimental results demonstrate the effectiveness of the proposed framework, which yields a TPR of 91% and a receiver operating characteristic (ROC) curve of 97%.

Ren et al. [84] introduced a new way of identifying DeFi attacks by concentrating on adversarial contracts instead of malicious transactions to identify possible attack intentions. The authors reported that most malicious contracts pursue comparable patterns, such as closed sources, anonymous fund sources, and frequent token-related function calls. Because of this finding, the authors were motivated to create a machine learning model to identify malicious contracts. The authors constructed a binary class dataset comprising 269 malicious and 13,000 normal contracts. They used this dataset to compare various machine learning classifiers. The best-performing classifier was LightGBM, which achieved an F1 score of 95.41% and a false positive rate of 0.15%.

Alkhalifah et al. [85] introduced a solution that calculates the participant’s contract and total balances and distinguishes the difference between them before and after any process in a trade that changes its state. This solution would enable developers to increase the security of smart contracts. The authors provided a proof-of-concept implementation that proves that the proposed method could present detection and prevention techniques to identify or eliminate reentrancy attacks while any smart contract is executed.

Yang et al. [86] proposed a new approach, BlockWatchdog, to identify reentrancy vulnerabilities once the attacker contract occurs. The attackers employ contracts to exploit vulnerabilities in blockchain technology automatically. The proposed approach can detect reentrancy vulnerabilities by analyzing attackers’ contracts and recognizing reentrant call flows. Additionally, new types of reentrancy bugs related to poor designs (e.g., when employing user-defined interfaces or Ethereum request for comment (ERC) tokens) can be detected via the proposed method. The authors implemented the proposed technique via cross-contrast static dataflow mechanisms related to cyberthreat logic captured from a real-world study that inspects malicious contracts deduced from 281 attack occurrences. They validated their method via 421,889 Ethereum contract bytecodes and detected 113 malicious contracts launched toward 159 victims. These malicious contracts caused losses to investors of approximately USD 908.6 million. This tool is promising compared with existing reentrancy detection tools, which detected only 18 out of 159 victim contracts.

Li et al. [87,88] presented a new tool known as ReDefender, which is capable of detecting reentrancy attacks on the basis of fuzz testing. The proposed method comprises four phases: contract preprocessing, fuzzing input generation, real-time information collection, and log execution analysis. In the first phase, once the contract is uploaded, the source code of that contract is fed into a preprocessing capability to infer the candidate pool for fuzzing. In the second phase, the fuzz engine generates fuzzing input. In the third phase, real-time information is collected with the help of a constructed agent contract capable of interacting with all contracts, including malicious contracts. This real-time information is captured during every fuzzing input execution. The last phase analyzes the captured information log to identify reentrancy attacks. The authors evaluated their technique via 204 tagged smart contracts, and their method achieved reasonable results in terms of accuracy and false positive rates. They also found four new reentrancy bugs when they applied their method to 395 on-chain contract accounts with more than 1,000 exchanged transactions.

Samreen et al. [89] proposed a hybrid approach that integrates static and dynamic analysis to identify Ethereum smart contracts’ reentrancy vulnerabilities. The proposed method customizes an attacker contract based on the specifications of the smart contract application binary interface (ABI). This customization aims to analyze the contract interaction to identify reentrancy vulnerabilities accurately. Based on the authors’ preliminary experiments, the technique detected the reentrancy vulnerability in five modified smart contracts. The proposed method can statistically analyze smart contracts to recognize possible vulnerable functions and utilizes dynamic analysis to ensure reentrancy vulnerability accurately. The experiments showed promising performance and a low false positive rate.

The basis of DeFi applications, which are smart contracts, are susceptible to various vulnerabilities that can be exploited by attackers, such as reentrancy attacks in which an illicit contract invokes a given function frequently before completing its execution. For example, the well-known DAO attack exploits the reentrancy vulnerability, which causes a loss of more than USD 60 million in Ethereum. These attacks can be detected/prevented using one of the deep learning-based approaches analyzed in this section and performing statistical analysis to recognize potential security vulnerabilities. Most code exploit detection approaches concentrate on identifying flash loan attacks and vulnerabilities that target smart contracts. FlashSyn effectively captures known vulnerabilities and malicious behaviors, while supervised learning methods detect attack types through transaction patterns. Moreover, approaches concentrating on adversarial contract patterns illustrate the necessity of continuous monitoring for suspicious behavior across multichain platforms to deal with exploit spread. Many current approaches are incompatible with unified blockchain security frameworks. Thus, it is essential to implement real-time detection applications that combine dynamic and static analysis for proactive attack detection.

### 6.3. Oracle Manipulation Detection

This subsection presents the approaches to identifying oracle manipulation, and Table 4 compares these techniques. To respond to an oracle manipulation attack, Arora et al. [90] analyzed the threat by normalizing the protocols for loanable funds (PLFs) attack models and standard operations. After the analysis step, the authors proposed a practical and robust technique to prevent oracle manipulation attacks effectively. The proposed method tracks each crypto asset price state, which consists of the current price and the timestamp attached to the recent update. Once the price constraints are imposed on oracle price usage, the proposed method ensures that the protocols for loanable funds can only employ a price oracle if the recently logged price falls within an identified threshold, eliminating the profitability of possible attacks. The authors then utilized historical market data to evaluate the proposed method. They found that it provides arbitrage attack prevention (which has been identified owing to minor price deviations) with high confidence. The proposed method has many advantages, including proactive protection from oracle manipulation attacks, ease of implementation, an oracle-agnostic mechanism, and cost and resource efficiency.

Price oracle manipulation attacks (POMAs) can be identified by analyzing single-transaction attacks, in which a threat is fully included in one transaction. However, Xi et al. [91] analyzed POMAs in blockchain technology (particularly in Ethereum) and reported that POMAs can spread into various transactions. Therefore, the authors presented a framework to identify POMAs that can outstretch multiple transactions. The framework uses generic rules to detect POMAs instead of relying on past attack patterns. The authors initially developed first-principle rules to determine POMAs similar to traditional stock market manipulation attacks. They then introduced a mechanism that utilizes these rules to identify POMAs that propagate through single and multiple transactions. This mechanism employs familiar features of POMA attackers’ demeanors to adjust its identification capability. The framework is evaluated via blockchain transactions collected over 2.5 years and a dataset assembled from the Code4rena audit reports. The experiments show that the proposed framework’s effectiveness in detecting 6.5X POMAs is greater than that in previous works. Additionally, the proposed framework outperformed previous methods, yielding a 1% worst-case false positive rate while achieving zero false negatives.

Deng et al. [92] proposed a method that automatically investigates the behavior of DeFi protocols if they incur an outlier oracle input. The proposed method employs a symbolic analysis using a given contract and builds a model consisting of constraints. The authors subsequently utilized a satisfiability modulo theory (SMT) solver to specify parameters that permit the safe operation of the proposed technique. Smart contracts utilizing oracle values are also given guard statements to mitigate oracle manipulation attacks. The experiments demonstrated that the proposed technique can efficiently analyze various ranges of DeFi protocols. The authors also evaluated previous approaches and reported that most of them utilize insufficient parameters to ensure security when exposed to significant oracle deviations.

Wang et al. [93] proposed a scalable security analysis method capable of identifying price oracle vulnerabilities before attacks occur. The main principle of this method is its ability to locally emulate price oracle attacks by transforming the data required for price calculation. The proposed method explores transactions to rebuild potential DeFi use patterns, thus substantially decreasing the number of simulation runs needed. The framework creates a report that includes the identified vulnerabilities to enable further analysis. The proposed method was evaluated via common and new price oracle vulnerabilities in DeFi protocols, in which five out of six common vulnerabilities and 27 new vulnerabilities were identified.

Wang et al. [94] presented a real-time attack detection system utilized to help identify threats targeting DeFi projects on Ethereum. The proposed system has two main capabilities. The first enables the system to identify vulnerable DeFi projects with the help of an automatic security analysis process. This analysis process implements symbolic reasoning via the traffic of significant service states, such as checks and asset prices, to ensure that outsiders cannot manipulate them. The second capability is a mechanism that monitors transactions and is installed off-chain to scan vulnerable DeFi projects. A thorough security analysis is conducted using transactions that are transmitted not only to that insecure DeFi project but also to other associated projects. The system searches for violations; if a violation occurs, the transaction that triggers this violation is flagged. The authors deployed their system in various DeFi projects and identified several security attacks that had not been discovered before.

Wu et al. [95] proposed a technique capable of detecting two new attacks and indirect and direct price manipulation threats. In the direct manipulation threat, the adversary directly manipulates the token price by using a vulnerable DeFi project to execute an unwanted trade. On the other hand, in the indirect manipulation threat, the adversary indirectly manipulates the token price that belongs to the vulnerable DeFi project, such as a lending app. To address these threats, the authors introduced a platform-independent mechanism to deduce high-level DeFi semantics by using raw Ethereum transactions to create a cash flow tree. The authors subsequently elevated low-level semantics to high-level semantics such as liquidity mining, liquidity cancelation, and token trading. The authors then identified price manipulation threats by employing the deduced DeFi semantics patterns. The proposed technique was evaluated on more than 350 million transactions, and 432 new attacks were identified. The identified new attacks were confirmed to belong to five zero-day attacks and four well-known security incidents.

A serious challenge encountered in the Web 3.0 ecosystem is oracle manipulation. As it links off-chain with on-chain data, oracle manipulation introduces vulnerabilities that hackers can exploit to modify asset prices or cause suspicious smart contract behaviors. Existing approaches believe decentralized oracles can offer resilience by combining data from various sources. However, these approaches are susceptible to Sybil attacks presented by distributed data providers. Furthermore, outlier detection approaches concentrating on identifying rapid price changes can proactively prevent manipulation attempts. Future work in oracle manipulation detection can investigate incorporating supervised learning capable of adapting to dynamic market conditions and emphasizing real-time oracle data integrity. Another research area could be introducing cross-chain oracle detection solutions that promote resilience against manipulation in linked DeFi networks.

### 6.4. Wash Trading Detection

Wash trading detection in DeFi is very challenging because of the absence of clear signals and reliable ground truth [96]. Graph and statistical techniques are utilized to detect wash trading with the help of expert-derived rules. Scholars have used statistical analysis to determine and investigate the impact of wash trades on exchanges and other DeFi markets as shown in Table 5. Cong et al. [97] introduced the first systemic technique to identify rouge cryptocurrency exchange transactions via robust behavioral and statistical regularities linked to legitimate trading. The data collected include 29 centralized exchanges, with regulated exchanges exhibiting transaction patterns similar to those observed in financial markets and nature. On the other hand, unregulated exchanges exhibit anomalous first significant digit distributions, transaction tail distributions, and size rounding, suggesting that prevalent manipulation most likely does not originate from exchange heterogeneity or a given trading strategy. The authors then utilized each unregulated exchange to quantify wash trading, finding that it exceeded 70% of the reported volume. The authors then explained how these faked volumes (i.e., estimated by trillions of dollars annually) enhance exchange rankings and temporarily distort prices related to exchange characteristics, such as the user base and age, associated with market circumstances and regulations.

The rapid growth of the NFT market has attracted the attention of numerous investors. Hence, it is necessary to conduct further research into the price variations in NFTs. There is little research on the use of NFT sales price fluctuations to predict possible deceptions via machine learning. Wang et al. [98] introduced a machine learning-based approach to predict NFT price fluctuations utilizing information obtained from the OpenSea marketplace. The authors collected three groups of data via a Python script: collection information, NFT information, and related account information. Then, they tested two well-known ensemble methods, AdaBoost and random forest, to predict the sale price and fluctuation by employing these regression and classification algorithms. The related account information (particularly the favorite number and creator activity status) enables machine learning models to perform accurate predictions. For regression purposes, on the one hand, AdaBoost provided a better prediction in determining the NFT sale price, with a root mean square error (RMSE) of 0.047. On the other hand, random forest was more accurate than AdaBoost in predicting price fluctuations, with an AUC of 0.956. The authors also suggested that investors should be more cautious and review the NFT creator’s information before taking serious action.

The NFT market lacks proper regulations; thus, serious cybercrimes such as fraud, money laundering, and wash trading might emerge. This deficiency in industry-wide regulations and the high presence of retail investors and amateur traders in the NFT market creates opportunities for cybercriminals to exploit vulnerabilities and carry out fraudulent activities. Thus, exploring and clarifying NFT trading cybersecurity issues is necessary. Song et al. [99] discovered common fraudulent activities such as wash trading that deceive legitimate traders. The authors collected data from the NFT market. They extracted quantitative attributes from the network, temporal views, and monetary information to train an unsupervised machine learning technique to categorize traders into clusters. The authors utilized K-means as a technique to classify traders. The clustering results are promising, and the authors discuss how proper regulations can decrease the potential for cybercriminal activities. The main advantage of this research is its ability to enable officials who develop NFT market regulations to narrow down bad actors’ search space and introduce perspicuity for amateur traders to defend themselves from upcoming suspicious activities.

Determining the factors affecting the performance of the NFT market is important but difficult because of the large amount of multimodel and heterogeneous NFT transaction data, including social media texts, numerical trading data, and images. Cao et al. [100], among the few researchers to address these issues to enable user analysis, proposed a system known as NFTTeller (i.e., an interactive dual-centric visual analytics system). Five domain experts assisted the authors in filtering dynamic and static impact features and gathering suitable data. The authors then created six analysis phases and designed NFTTeller to introduce the development of NFT transactions and integrate NFTs’ market performance with impact features. Remarkably, the authors designed an augmented chord chart that contains a radial stacked bar graph to investigate intersections between NFT collection projects and whale accounts.

One limitation when validating wash trading detection approaches is the lack of a publicly available dataset, so these approaches rely heavily on custom datasets and descriptive analysis, as opposed to money laundering detection, where public-labeled datasets such as Elliptic are available to enable fair performance comparisons. This absence of a public dataset highlights the necessity for distributable data and standardized measures to benchmark wash trading approaches effectively. Statistical analyses indicate that over 70% of transactions on unregulated exchanges may involve wash trading. Clustering methods, like K-means, can identify traders displaying suspicious behavior. However, when employing these approaches in large or heterogeneous environments like DeFi, these techniques will encounter challenges related to scalability. Some applications, such as NFTTeller, introduced an integrated perspective that binds trading information with external impact characteristics like social media data. A hybrid method that utilizes machine learning and statistical analysis might present effective recognition features, particularly in rapidly emerging markets such as NFTs.

### 6.5. Money Laundering Detection

This subsection presents the approaches that detect money laundering in the DeFi paradigm, and Table 6 compares these approaches. Alarab et al. [101] investigated the effectiveness of graph convolutions integrated with linear layers to detect money laundering in blockchain networks. The authors combined node embeddings received from graph convolutional layers, each designated with a hidden layer. Each hidden layer originates from the node feature matrix linear transformation and follows the multilayer perceptron. The proposed method was tested on the Elliptic dataset [102] and compared with baseline results introduced with the dataset, which achieved acceptable accuracy, precision, recall, and F1 score results.

Lorenz et al. [103] claimed that even though supervised learning algorithms achieved decent results in detecting antimoney laundering in cryptocurrencies, they should be trained on a large amount of training data, which are not currently available. Thus, the authors introduced an active learning approach that assumes minimal access to labeled data. The authors also tested several unsupervised anomaly detection methods and reported that they are inadequate for detecting suspicious patterns in a Bitcoin transaction dataset. The proposed active learning approach showed promising results comparable to those of the supervised baseline when only 5% of the label data were utilized. The main advantage of this technique is that it is realistic because, in real-life situations, only limited data can be obtained by experts through manual annotation. Thus, using a limited number of data mimics the typical normal and suspicious activities that appear in cryptocurrencies.

Hyun et al. [104] introduced a multirelational graph neural network (GNN) approach that combines the edges of orientation and characteristics such as transaction frequency and amount to detect money laundering in cryptocurrencies. The authors also presented an adaptive neighbor sampler to enhance finding performance and reduce computational expense. The authors validated their framework on public datasets, which proved its effectiveness compared with state-of-the-art GNN-based approaches used to detect fraud.

Badawi et al. [105] compared the performance of two well-known machine learning algorithms in detecting cryptocurrency antimoney laundering and learning data patterns. The compared machine learning algorithms (i.e., decision trees and shallow neural networks) were tested on the Elliptic dataset. The comparison analysis revealed that the decision tree achieved an accuracy of 93.4%, which outperformed the shallow neural network, which yielded an accuracy of 89.9%.

Zhou et al. [106] outlined data analytical prerequisites for transaction supervisors and investigated cryptocurrency transaction domain knowledge regarding anti-money laundering. The authors also introduced a visual analysis method to detect illegitimate money laundering users and a multiview user interface that illustrates the algorithm results and related transaction data from a visualization perspective. The authors developed a visualization approach at the interface motivated by an abacus to envision cryptocurrency transaction patterns, enabling supervisors to locate money laundering indications and infer the launderer’s trading tactics. The algorithm’s performance is evaluated via real-world data to demonstrate the effectiveness of the proposed method.

Liu et al. [107] presented an enhanced graph embedding technique to detect money laundering in cryptocurrency. The proposed method can thoroughly learn money laundering intruders’ behavioral patterns and transaction networks’ structural data. Furthermore, it can extract relevant features in real time to identify addresses for money laundering. The authors merged the gas price and timestamp from transaction records into a new weight and modified the return and exploration parameters to modulate the random walk’s sampling tendency, distinguishing the nodes involved in money laundering. The proposed technique was validated via a customized dataset constructed from real Ethereum data and tested via various machine learning algorithms, such as random forest, support vector machine (SVM), naive base, XGBoost, and logistic regression. Compared with other graph embedding techniques, the proposed method was effective at extracting money laundering account features.

Yang et al. [108] proposed a two-tier framework to detect virtual currency money laundering. The framework combines heuristic rules and learning modalities. The authors examined statistical risk distribution features and transaction instances to create heuristic rules. Using various transaction datasets, the authors merged long short-term memory (LSTM) and a graph convolutional neural network (GCNN) to effectively identify abnormalities that represent money laundering patterns. The integrated learning method uses a hard voting scheme to strengthen the identification of money laundering methods by fusing several anomaly detection algorithms, such as isolation forest and histogram-based outlier scoring (HBOS). This method’s advantages include detecting money laundering activities through unlabeled samples, explicitly creating outlier transaction data, and implicitly identifying suspicious transaction data that heuristic rules cannot capture. The proposed method makes acquiring labeled data for antimoney laundering detection easier while improving the accuracy and generalizability of unsupervised learning methods for recognizing money laundering activities.

Alarab and Prakoonwit [109] also introduced a hybrid model that merges LSTM and a GCN called the temporal GCN and is used to detect money laundering transactions. The proposed method was evaluated on the Elliptic dataset. The authors utilized an active learning technique on a vast Bitcoin transaction graph dataset. The authors employed both Monte Carlo-based adversarial attack and Monte Carlo dropout (i.e., these two methods are Bayesian approximations) to address uncertainties when applying active learning. The authors compared the temporal GCN method with previous methods on the same dataset and used the same experimental settings and reported that their proposed method outperformed the other methods. They also compared the performance of the two Bayesian approximation techniques with the baseline random sampling model, which achieved decent results compared with the baseline sampling model.

Lo et al. [110] introduced a GNN-based framework utilizing both a self-supervised graph isomorphism network (GIN) and self-supervised deep graph infomax (DGI) along with a random forest supervised learning algorithm to identify money laundering activities in cryptocurrency. The authors utilized the Elliptic dataset to evaluate their proposed framework, which yielded reasonably good results compared with the state-of-the-art results in terms of four performance metrics: precision, recall, F1 score, and area under the curve (AUC). The proposed framework confirms self-supervised GNN prospects in identifying suspicious activities in cryptocurrency networks.

Kolesnikova et al. [111] proposed a stacked machine learning approach to detect money laundering in virtual currencies. The proposed method consists of a random forest and a graph convolutional network. The experimental results show the existence of potential shadow transactions of approximately 2-3% to the market. With the evolution of the financial domain (particularly the emergence of cryptocurrency), a new generation of illicit transactions has emerged due to blockchain technology’s anonymity, which enables users to establish an unlimited number of wallets utilizing alias addresses. This anonymity makes it difficult to specify the actual users, enabling cybercriminals to make illicit transactions. Moreover, the blockchain stores all transaction information, which enables cryptocurrency organizations to obtain the necessary details to identify suspicious behavior patterns with the help of machine learning.

Yu et al. [112] presented a deep learning-based cryptocurrency money laundering detection approach. The framework consists of an encoder that identifies the importance of the surrounding transaction through the transaction flow amount values. The authors also employed a graph embedding technique and combined the original features and the features extracted from the graph embedding technique to address the problem of feature distortion. Then, they applied a graph autoencoder to obtain the prevailing structural information of different transactions. The detector is the concatenated embedding utilized to produce the classification results. The framework also introduced a mutual learning capability that benefits from structure reconstruction and transaction classification losses. The proposed method was validated using the Elliptic dataset, outperforming the state-of-the-art techniques.

Wu et al. [113] introduced a multiclass dataset to overcome the issue that the only public dataset (i.e., the Elliptic) has, which includes two classes (licit and illicit transactions) and does not comprise all money laundering behaviors in the thriving cryptoasset market. The authors presented a framework that determines money laundering addresses initiated from Ethereum heist occurrences and constructed a multiclass dataset. The authors surveyed the source cryptocurrency cyberattackers’ accounts, such as Defi exploiters, scammers, and exchange hackers. They subsequently utilized the taint analysis mechanism to trace diverse downstream transactions and address them step by step. Then, they determined and classified service providers and explored advanced money laundering techniques such as counterfeit token creation and token swaps. The experimental analysis revealed interesting findings regarding cryptoasset money laundering, highlighting the increase in money laundering techniques such as establishing fake tokens and posing as speculators.

The money laundering detection methods range from supervised machine learning to graph-based anomaly approaches. On the one hand, the problem with supervised machine learning methods, such as neural networks and tree-based approaches, is that they require large amounts of labeled data rarely available in real-world scenarios. On the other hand, the GNN-based and other unsupervised learning approaches are practical in analyzing the patterns of blockchain transactions. However, they are not precise like the supervised learning approaches when sufficient labeled data are available. Therefore, integrating active learning to decrease the dependence on labeled data while achieving decent performance is decent. Moreover, some visualization approaches, such as the abacus-based technique, could be combined with other detection methods to offer unique investigation interfaces and improve the explainability and effectiveness of the money laundering detection approaches.

### 6.6. Network Threat Detection

This subsection investigates the techniques that detect the attacks that target the network resources, and Table 7 compares these methods. Swathi et al. [114] noted that blockchain is vulnerable to traditional attacks because of the lack of robust identity management. The authors revealed that permissionless/public blockchain is particularly susceptible to Sybil attacks in which intruders construct numerous pseudonymous identities, creating rogue user accounts and forcing legitimate nodes into the minority. The created virtual entities pretend to be legitimate to establish a disproportionately large effect on the blockchain environment. This attack could be the first attack to be launched, followed by other effective attacks such as DoS and DDoS. The authors explored the impact of a Sybil attack on blockchain networks, especially its notable effect on system throughput. They presented a solution that allows nodes to monitor the behavior of other nodes and inspect those nodes that transfer blocks for a specific user. These nodes are swiftly determined and put on a blacklist, and notifications are sent to other nodes in the blockchain network to restrict Sybil attacks.

Peer-to-peer networks, including blockchain networks, are vulnerable to Eclipse and Syble threats. Unfortunately, existing techniques are insufficient for detecting these attacks because of the imbalanced datasets that they use to evaluate their techniques and discriminate incomplete feature declarations and prosaic feature perceptions. Geepthi et al. [115] introduced Sybil and Eclipse attack detection and mitigation techniques based on fuzzy secure Kademlia (FSK). The authors suggested that the node be authenticated through an infrastructure server by sending its MAC addresses, node angle (NA), location, and node residual energy (NRE). On the one hand, network traffic is monitored in real time. If the packet’s ID, NA, location, and NRE match the information recorded at the infrastructure server, the packet is classified as normal. If the incoming packet does not match the location recorded on the server, the packet is classified as an attack. Once the attack is identified, it can be easily located and removed. The proposed framework was compared with related methods, and its effectiveness in terms of energy consumption and detection time was demonstrated.

DDoS and Sybil attack detection approaches illustrate the significance of integrating conventional security mechanisms with decentralized measures. For instance, Sybil attacks can be mitigated by utilizing blockchain-based reputation systems and employing fuzzy learning detection approaches to identify DDoS attacks in real time. However, these approaches can omit the interconnection between network and application layer threats. Future work can focus on incorporating novelty detection mechanisms across the layer stack to introduce a complete protection framework against attacks that might propagate through multiple layers.

### 6.7. Web 3.0 Security Breaches and Detection Methods Case Studies

As seen in well-known blockchain data breaches, web 3.0 security challenges have evident real-world implications. For example, the 2016 decentralized autonomous organization (DAO) attack exploited a reentrancy vulnerability in a smart contract, causing a loss of USD 60 million in Ethereum [116]. So, introducing improved auditing tools for smart contracts is necessary to prevent similar security breaches. Current detection techniques (i.e., those based on GNN) can proactively identify vulnerabilities. For example, GNN-based approaches can monitor transaction flows and recognize patterns to detect rentrancy attacks early.

In 2021, another security breach targeted a DeFi platform called Poly Network, exploiting the cross-chain vulnerability [117]. This hack enables the attackers to steal USD 610 m of digital assets. This attack clarifies the current vulnerabilities in cross-chain protocols and highlights significant security issues in DeFi platforms. Therefore, it is essential to apply more secure cross-chain interoperability solutions. To prevent this attack, existing solutions proposed off-chain and on-chain monitoring systems and methods capable of tracking real-time transactions.

The case studies demonstrate the de facto alignment between the theory and practical state-of-the-art detection techniques. It also highlights how these approaches can mitigate attacks that emerge in the Web 3.0 paradigm. By investigating real-world case studies, we can deduce how these state-of-the-art methods can improve security and offer suitable solutions to the issues that appear in emerging digital environments such as Web 3.0.

## 7. Challenges and Opportunities

The most distinguishing features of Web 3.0 are its ability to be deployed in a distributed internet infrastructure, its user-centered nature, users’ ability to identify themselves autonomously, personal data privacy, and equality of rights for users and developers. However, Web 3.0 presents various challenges that can be classified into underlying blockchain infrastructure issues, digital asset and smart contract challenges, and application-specific issues.

### 7.1. Underlying Blockchain Infrastructure

Web 3.0 users may encounter a high volume of frequent transactions from accounts under their control. This traffic increases the difficulty of identifying entities operating these accounts, leading to various anonymous transactions and insecure processes. Machine learning approaches could be utilized to provide de-anonymity, but these approaches might present other issues related to user privacy breaches. Many logs related to user traces and activities can be collected via Web 3.0. Because these logs are derived from the public blockchain, accumulating traces over time may lead to potential privacy disclosure issues for users. More specifically, because of blockchain’s transparency and openness, DeFi protocol market states are observable online and in real time. Transactions exchanged between users are deployed on the blockchain network and stored on a public platform (e.g., a Bitcoin mempool). Users can access the transactions stored in this public platform before mining them in a block. Thus, attackers can predict future market states via non-executed transactions to manipulate the market. For example, attackers can observe a targeted transaction on a public platform and backrun or frontrun that transaction to earn returns.

Additionally, because Web 3.0 applications consist of smart contracts running on a blockchain system, they are exposed to potential vulnerabilities, such as eclipse attacks [118] that target blockchain could divide the network, leading to conflicting logic among various transactions [119]. Consensus vulnerabilities may arise from block reorganization [120] and feather forking [121], potentially leading to double spending issues in DeFi networks. Moreover, blockchain-limited throughput might increase the chance of network congestion, leading to DeFi transaction delays.

To detect security issues related to DeFi protocols effectively, it is necessary to first launch applications used in the targeted transactions. Hence, these applications might be affected by a security breach. These security problems are associated with DeFi networks and consistently affect users in the long term [122]. Thus, increasing users’ awareness and mitigating security risks in DeFi networks are important.

As we discuss in the following subsections, two more hot research areas that need to be further enhanced are privacy and oracle manipulation issues.

#### 7.1.1. Privacy

The most understudied research area in DeFi is privacy and anonymity [123,124]. A standard view is emerging: DeFi protocols can be mainstream only once the privacy problem is handled. Owing to the highly sensitive nature of financial data, privacy is paramount to everyone. In particular, DeFi users desire confidential transactions that cannot be exposed by unauthorized parties, who might reveal the financial information that users exchange. Hence, Web 3.0 should offer unconditional privacy and anonymity for trading crypto assets. Unfortunately, at present, most DeFi transactions are Ethereum dependent and are exchanged between users where agents have pseudoanonymity [125]. This implies that if a trader’s identity can be linked to an on-chain address, all activities performed using this address by the trader can be tracked. Although the current state-of-the-art solutions in multiparty computations [126,127] and zero-knowledge proofs [128,129] are promising, these techniques need to be deployed in a real environment to prove their effectiveness. The main challenge of these approaches is the high computational cost, which might prevent them from being deployed in real DeFi environments. Thus, a research opportunity could be to introduce a privacy-preserving technique that has a low computational cost so that the underlying blockchain can be efficiently deployed in DeFi environments.

#### 7.1.2. Oracle Issue

An oracle, which is provided to the DeFi environment, is a third-party service that allows smart contracts in the blockchain network to access external and off-chain resources such as token rates and price information [130,131]. Although the oracle plays an integral role in the DeFi paradigm, it presents some security threats. Additionally, the underlying capabilities of the oracle used in the DeFi ecosystem are ambiguous and securely unanalyzed. These capabilities and deployment procedures, such as the volume of price updates and the aggregation of price values gathered from multiple nodes, are not trackable and transparent, exposing them to cyberattacks. Additionally, the level of trust acquired by the oracle is not clear, and most likely, the majority of traders are not aware of it. Moreover, the potential impact of a malicious oracle (or several oracles) has not been explored in the DeFi paradigm.

Although communication between two on-chain entities is straightforward, conveying data from external entities, such as a given website, to an on-chain party (e.g., a smart contract) comes with its own challenges. Various DeFi applications depend on external data, such as price information and exchange rates; hence, DeFi oracles offer this information. Because the data are generated by DeFi oracles, there is an impact on users and smart contracts if the DeFi oracle is not trusted; thus, conveying external information to on-chain entities is a major concern. In particular, DeFi application secrecy is dependent on the accuracy, reliability, and correctness of the data transferred from the oracle.

### 7.2. Digital Assets and Smart Contracts

Smart contracts enable digital assets such as fungible tokens, NFTs, and stablecoins to be exchanged via a trading platform. Moreover, regulations and policies already govern blockchain cryptocurrencies such as Ethereum and Bitcoin; thus, the regulations of cryptocurrencies and other Web 3.0 applications must align. Like conventional programs, smart contracts could include vulnerabilities; hence, smart contracts (i.e., holding more than a trillion dollars worth of virtual coins) are important and have the potential to be targeted by cybercriminals. Several vulnerabilities in smart contracts can be mined by attackers, which leads to considerable losses [132,133]. Many proposals have been introduced in industry and academia to safeguard against smart contract vulnerabilities [134,135]. Most current approaches that attempt to patch or detect smart contract bugs depend on static analysis and fuzzing methods. These methods fail to efficiently detect or mitigate attacks targeting smart contracts’ vulnerabilities because of the composable nature of smart contracts (i.e., those that produce abnormal behaviors). Additionally, these techniques cannot effectively address incidents in which a problem might occur because of a change in the external entity of smart contracts, such as a sudden change in a virtual coin price retrieved from the oracle. In this subsection, the limitations of current approaches are explained, which can be categorized into two classes: shortcomings of deep learning-based approaches and weaknesses of traditional machine learning techniques.

#### 7.2.1. Shortcomings of Deep Learning-Based Approaches

Deep learning-based approaches require many vulnerable contracts to train a reasonable neural network model and create an effective detection model. However, the biggest issue is the lack of a sufficient smart contract vulnerability dataset; thus, the performance and credibility of these approaches are hindered. Furthermore, the lack of interpretability in deep learning techniques is exacerbated by the black-box nature of neural networks. Training the deep learning model on normal behavior via anomaly detection with autoencoders or generative adversarial networks, for example, and identifying anomalies that deviate from that behavior, are promising research topics.

#### 7.2.2. Traditional Machine Learning Techniques

Compared with deep learning-based approaches, traditional machine learning techniques perform poorly. However, they might be a good option when there are not enough vulnerable contracts to train the model. Additionally, owing to the lack of a sufficient dataset of vulnerable smart contracts, applying an anomaly or novelty detection model such as a one-class SVM or isolation forest and training it via normal smart contracts is promising. Moreover, explainable AI could be applied not only to detect vulnerabilities but also to explain the type of detected vulnerability.

### 7.3. Network Threats and Compound Attacks

Although Web 3.0 provides secure decentralization and privacy measures, several security issues remain open. Future research could focus on how light deep learning models can be employed to identify Web 3.0 threats such as Sybil attacks, the approaches that can be introduced to continually learn the behavior of DeFi traffic and adapt to new abnormal behavior, and the methods that should be developed to present explainability and identify the type of the detected attack. New technical approaches could integrate federated learning with decentralized identity systems to create a collaborative attack detection framework that leverages blockchain technology while preserving user privacy. Furthermore, due to the scarcity of real-time anomaly detection methods in the surveyed solutions, it is preferable to present a new anomaly detection technique utilizing edge computing to combat weaknesses in IoT devices that could be linked to Web 3.0. These ideas must enhance Web 3.0 infrastructure by implementing proactive and scalable defenses against emerging threats. These approaches are essential for ensuring a secure and robust Web 3.0 environment. Future work could concentrate on mitigating compound attacks by leveraging holistic security frameworks that rely on the interplay between multiple layers of Web 3.0 architecture. Approaches like cross-contract vulnerability analysis and cross-layered outlier detection would be significant for detecting and preventing compound attacks.

## 8. Conclusions

The emergence of Web 3.0 signifies a new phase for the internet, emphasizing distributed technology that prioritizes data ownership and value expression. Its main goal is to give users control and ownership of their data and digital assets, moving away from the power of large corporations. Web 3.0 relies on decentralization and uses blockchain technology to ensure secure user communication. However, it still faces security challenges that could hinder its deployment and expose user data and digital assets to cybercriminals. This survey explores the current evolution of Web 3.0, presenting its background, foundation, and applications. It also provides an overview of cybersecurity risks associated with Web 3.0 mature application domain (i.e., DeFi), categorizing them based on their Web 3.0 layer architecture; additionally, it investigates and categorizes the state-of-the-art efforts to address these threats based on their security risks. Finally, it offers insights into potential future directions for Web 3.0 security. While this survey concentrates on DeFi ecosystems, the findings and insights can be broadly adapted to other Web 3.0 application domains. In future work, we will investigate how the presented cybersecurity challenges impact other application domains, such as healthcare, in which the confidentiality of patient’s health records is crucial, and the metaverse, in which virtual identities and assets are increasingly valuable.

## Figures and Tables

**Table 1:** The main characteristics of Web 2.0 and Web 3.0.

	Web 2.0	Web 3.0
**Payments**	Commercial banks host online bank transfers between beneficiaries; e-money providers such as PayPal and Alipay (in China) are utilized as digital payment platforms and payments are collected in existing currencies [8].	The spread of cryptocurrencies, the era of direct payments between web users without the existence of mediators; the currency is not owned by the central bank and is not collateralized [9].
**Financial Services**	Financial services are not integrated into Web 2.0; they are accessible via web-based e-commerce sites in Web 2.0 paradigm.	Financial services are one of the building blocks of Web 3.0 represented by well-known blockchain-based technologies such as DeFi [10,11].
**Identity Management**	It relies on key public infrastructure to manage identities; also, personal identity is proofed by establishing specific routines and cloud-based identity.	It manages digital identities and allows individuals and businesses to have full ownership to control their accounts and personal data (i.e., Self-sovereign identity) [12].
**Data Ownership**	Companies host data and utilize them for different purposes.	Users own data and utilize them for their own purposes.
**Trust Anchors**	Central authorities and large companies.	End users employing consensus protocols [13]
**Protocol Characteristics**	Protocols attach isolated applications and automate data transmission ignoring how the data are stored (i.e., stateless) [14].	Decentralized computing in stateful manner in with universal state is collectively maintained [14].
**Buisness Models**	Large social media companies that such as Alphabet, and Meta and giant e-commerce companies such as Amazon (i.e., companies that commercialize customers data).	Decentralized autonomous organization (DAO) [15] and decentralized finance (DeFi) models that provide decentralized payment services, contracting, and fundraising [16].
**Famous Use Cases**	Social media networks and e-commerce.	non-fungible tokens (NFTs) [17,18], which symbolize real-world things such as real estate and artwork or can exemplify identities for individuals and property rights, DeFi, and healthcare applications that utilize blockchain.

**Table 2:** Comparison between the SCAM detection approaches.

Reference	Description	Technical Detail	Remarks
Onu et al. [43]	Developed a machine learning-based method to identify Ethereum Ponzi schemes.	After comparing three machine learning algorithms, they found that the random forest outperformed the others with a class score of 88.33% and an accuracy of 94%.	Also reduced the number of features from 70 to 10 to improve detection time while maintaining high accuracy.
Vassallo et al. [72]	Proposed a method for detecting malicious activities in crypto infrastructures, including Ponzi schemes and scams.	The approach, Adaptive Stacked eXtreme Gradient Boosting (ASXGB), outperformed other methods when tested on transaction and account levels.	Synthetic Minority Over-Sampling (SMOTE) and Neighborhood Cleaning Rule (NCL) data sampling techniques improved transaction level recall.
Xiang et al. [73]	Developed a new dataset and tested it using classifiers to detect suspicious activities in crypto platforms.	The experimental results showed promising results in identifying cybersecurity threats targeting the Bitcoin network, achieving accuracy rates of 93% to 97%.	They also introduced a technique to analyze Bitcoin transaction graphs and studied behavioral patterns associated with Bitcoin addresses.
Li et al. [74]	The authors developed a method called Temporal Transaction Aggregation Graph Network (TTAGN) to identify scams on Ethereum.	They represented the Ethereum transaction network using temporal relationships between nodes and combined trading features to differentiate scamming addresses from legitimate ones.	The method outperformed state-of-the-art techniques, achieving an AUC of 92.8% and an F1 score of 81.6% on a real-world dataset.
Fu et al. [75]	Developed a new approach for identifying scams on crypto platforms using a graph neural network.	It includes a mechanism to process imbalanced data and measure transaction time. It extracts time features from the graph using attention and LSTM procedures. These time features are combined with baseline features and input into merged GCN and SMOTE models to detect phishing attempts.	The method was tested on a custom dataset and demonstrated promising results, achieving an accuracy of 97.22% and an AUC of 96.67%.
Elmougy et al. [76]	A new holistic applied artificial intelligence approach was introduced to identify fraud in the Bitcoin network.	Presented an extended version of the ELLiptic dataset, called ELLiptic++, comprising 822,000 Bitcoin wallet addresses with 56 features and 1,270,000 temporal interactions. Using four categories of graph data, they detected fraud in crypto platforms and executed fraud detection schemes using machine learning techniques.	The results show the effectiveness of adding improved features in detecting illicit addresses and transactions, giving detailed comprehension of money laundering weaknesses for developing methods for fraud detection and mitigation.
Wahrstatter et al. [77]	Studied the Bitcoin user graph to identify suspicious accounts involved in illicit activities and introduced new features to detect suspicious behavior accurately.	They used an unsupervised machine learning method, specifically a clustering algorithm, to thoroughly analyze the network. It was applied to a Bitcoin user graph, customized for CoinJoin, to study the network at a user-centric level and enhance privacy.	It successfully identified most illegal activities within suspicious clusters, with accounts linked to suspicious behavior often demonstrating short paths to CoinJoin wallets and outlier behavior.
Garin and Gisin [78]	Compared different classifiers, including decision trees, random forests, gradient boosting, and logistic regression to detect suspicious activities in Bitcoin.	They balanced the classes, optimized the hyperparameters, and used active learning to enhance performance. They applied a feature selection method to identify the most important features and decrease the detection time.	The comparative results indicated that the gradient-boosting outperformed the other methods based on various evaluation metrics.
Aziz et al. [79]	Proposed a deep learning-based approach using a unique optimization strategy to detect suspicious transactions in Ethereum.	The framework combines deep learning with an optimized genetic algorithm-cuckoo search (GA-CS).	Their approach achieved better accuracy than well-known supervised machine learning algorithms, including random forest models.
Nayyer et al. [80]	The researchers used a combination of machine learning classifiers to detect suspicious Bitcoin activities.	They employed four well-known classifiers, namely, naive Bayes, KNN, decision tree, and random forest in an ensemble method and fine-tuned the model using various methods.	The proposed framework achieved 96% precision, 98% recall, 97% F1 score, accuracy, 99% AUC-ROC, and 3% FP.
Pragasam et al. [81]	A new method has been introduced to determine anonymity in crypto platforms like Ethereum.	This method uses multiclass machine learning algorithms to classify Ethereum’s externally owned accounts (EOAs) by analyzing transaction patterns and activity linked with account addresses.	The XGBoost classifier achieved the highest accuracy at 75%, followed by random forest at 73.7%, and gradient boosting at 73%.

**Table 3:** Comparison between the code exploit detection approaches.

Reference	Description	Performance	Remarks
Chen et al. [82]	The researchers developed an automated technique to investigate malicious transactions using flash loans in DeFi protocols.	Introduced numerical approaches to approximate DeFi protocol behaviors and used an optimization query to explore malicious behavior. They also presented a refinement method and utilized a tool called FlashSyn to implement their framework.	The framework was validated using vulnerable DeFi protocols, demonstrating effectiveness in identifying potential flash loan invasions.
Wang et al. [83]	Created a deep learning framework to detect and classify DeFi attacks, such as flash loan and price manipulation attacks.	The framework uses a neural network with a customized local model, a global model, and an ensemble mechanism to classify attacks.	After testing the framework with data from 50,910 DeFi transactions, it achieved a 91% true positive rate and a 97% ROC curve.
Ren et al. [84]	A new method for detecting DeFi attacks has been introduced.	Instead of focusing on malicious transactions, it identifies potential attack intentions by analyzing adversarial contracts. They observed that most malicious contracts exhibit similar patterns, including being closed-source, having an anonymous fund source, and making frequent token-related function calls. As a result, they developed a machine learning model for identifying malicious contracts.	They created a binary class dataset to compare different machine learning classifiers. The best-performing classifier was LightGBM, which achieved an impressive F1 score of 95.41% and a false positive rate of 0.15%.
Alkhalifah et al. [85]	Introduced a method for calculating a participant’s contract and total balances and distinguishing the difference between them before and after any trade process that changes its state.	This solution aims to help developers enhance the security of smart contracts.	Provided a proof-of-concept implementation that demonstrates how the method could be used to detect and prevent reentrancy attacks during the execution of any smart contract.
Yang et al. [86]	Proposed BlockWatchdog to identify reentrancy vulnerabilities in attacker contracts.	This approach detects reentrancy vulnerabilities, including new types of bugs through static dataflow analysis. The authors tested it on 421,889 Ethereum contract bytecodes and detected 113 malicious contracts, causing USD 908.6 million in losses to 159 victims.	Compared with existing tools, BlockWatchdog detected 113 out of 159 victim contracts, while existing tools only detected 18.
Li et al. [87,88]	They introduced ReDefender, a tool for detecting reentrancy attacks using fuzz testing.	The method has four phases: contract preprocessing, fuzzing input generation, real-time information collection, and log execution analysis. They evaluated the method with 204 contracts, achieving decent results.	They also found four new reentrancy bugs in 395 contract accounts with over 1,000 transactions.
Samreen et al. [89]	The researchers proposed using both static and dynamic analysis to identify reentrancy vulnerabilities in Ethereum smart contracts.	They customized an attacker contract based on the smart contract’s ABI specifications to accurately analyze contract interactions.	The method successfully detected reentrancy vulnerabilities in five modified smart contracts, showing good performance with a low FPR.

**Table 4:** Comparison between the oracle manipulation detection approaches.

Reference	Description	Performance	Remarks
Arora et al. [90]	Conducted an analysis to standardize operations for responding to Oracle manipulation attacks	They proposed a method to prevent these attacks. The method involves tracking cryptoasset prices and imposing constraints on Oracle price usage to eliminate potential attacks’ profitability.	It offers proactive protection from these attacks, is easy to implement, and is cost-efficient.
Xi et al. [91]	Analyzed POMAs in blockchain (especially Ethereum) and developed a framework to identify POMAs spanning multiple transactions.	It uses first-principle rules to identify POMAs to traditional stock market manipulation attacks. They propose a method that uses these rules to determine POMAs propagating through single and multiple transactions.	Unlike previous methods, their framework uses generic rules to detect POMAs, resulting in a 6.5X improvement in detection. It achieved a 1% worst-case FPR and zero FNR, outperforming previous approaches.
Deng et al. [92]	Developed a method to automatically investigate DeFi protocol behavior when encountering outlier oracle inputs.	This method uses symbolic analysis to build a model with constraints and employs an SMT solver to ensure safe operation. Smart contracts using oracle values are equipped with guard statements to prevent manipulation attacks.	The proposed technique efficiently analyzes various DeFi protocols and addresses the issue of insufficient parameters in previous approaches.
Wang et al. [93]	Proposed a security analysis method to identify price oracle vulnerabilities before attacks occur.	This method can locally emulate price oracle attacks by transforming the data used for price calculation, significantly reducing the needed simulation runs. It generates a report listing identified vulnerabilities for further analysis.	When evaluated, it successfully identified five out of six common vulnerabilities and 27 new vulnerabilities in DeFi protocols.
Wang et al. [94]	A real-time attack detection system has been created to identify threats targeting DeFi projects on Ethereum.	The system can identify vulnerable DeFi projects and monitor transactions to scan for security violations. The authors implemented this system in various DeFi projects and found previously undiscovered security attacks.	The authors deployed their system in various DeFi projects and identified several security attacks that had not been discovered before.
Wu et al. [95]	Developed a method to detect two new types of attacks—direct and indirect price manipulation threats—in DeFi projects.	Developed a platform-independent method to extract high-level DeFi concepts by analyzing raw Ethereum transactions and creating a cash flow tree. They then translate low-level transaction details into high-level DeFi concepts such as liquidity mining, liquidity cancellation, and token trading. They identified potential threats related to price manipulation by analyzing the patterns derived from the DeFi concepts.	They evaluated the technique using over 350 million transactions, identifying 432 new attacks, including five zero-day attacks and four well-known security incidents.

**Table 5:** Comparison between the wash trading detection approaches.

Reference	Description	Technical Detail	Remarks
Cong et al. [97]	Developed a method to detect suspicious crypto exchange by analyzing patterns related to legitimate trading on 29 centralized exchanges.	Regulated exchanges show similar transaction patterns to traditional financial markets, while unregulated exchanges exhibit abnormal transaction distributions, suggesting potential manipulation.	The study found that wash trading accounted for more than 70% of reported volume on unregulated exchanges.
Wang et al. [98]	Used machine learning to predict NFT price fluctuations by analyzing data from the OpenSea marketplace.	Tested two methods, AdaBoost and random forest, using regression and classification algorithms. While AdaBoost was better for predicting sale prices, random forest was more accurate for predicting price fluctuations.	Recommended that investors review NFT creators’ information before taking action.
Song et al. [99]	Identified fraudulent activities like wash trading in the NFT market.	Used unsupervised machine learning (i.e., K-means technique) to group traders and found promising results. They discussed how regulations could reduce cybercriminal activities.	This research helps regulators pinpoint bad actors and provides transparency for inexperienced traders.
Cao et al. [100]	Developed NFTTeller, an interactive visual analytics system to visualize diverse NFT transaction data.	Visualized data includes social media texts, numerical trading data, and images. They worked with five domain experts to filter dynamic and static impact features and gather suitable data. The authors then created six analysis phases and designed NFTTeller to track NFT transactions and link market performance with impact features.	They developed an augmented chord chart with a radial stacked bar graph to investigate intersections between NFT collection projects and whale accounts.

**Table 6:** Comparison between the money laundering detection approaches.

Reference	Description	Technical Detail	Remarks
Alarab et al. [101]	Studied the impact of integrating graph convolutions with linear layers to detect money laundering in blockchain networks.	Node embeddings are obtained from convolutional layers, each with a hidden layer. Each hidden layer comes from the linear transformation of the node feature matrix and is followed by the MLP.	It was tested on the Elliptic dataset and compared with baseline results from the dataset, showing acceptable accuracy, precision, recall, and F1 score results.
Lorenz et al. [103]	Proposed an active learning method to deal with the minimal availability of label data.	Found that anomaly detection methods were inadequate for identifying suspicious patterns in a Bitcoin transaction dataset.	Yielded promising results comparable to supervised baseline, with 5% of labeled data. In real life, experts have limited access to label data, so using a small amount of data reflects typical activities.
Hyun et al. [104]	Presented a multirelational graph neural network (GNN) approach to identify money laundering in cryptocurrency.	Integrates orientation edges and features such as transaction frequency. They presented an adaptive neighbor sampler to enhance finding performance and reduce computational expense.	Validated the framework on public datasets, showing its effectiveness for fraud detection compared with state-of-the-art GNN-based approaches.
Badawi et al. [105]	Thoroughly assessed two classifiers for detecting crypto money laundering and revealing complex data patterns.	The algorithms, namely decision trees and shallow neural networks were compared using the Elliptic dataset.	The decision tree achieved 93.4% accuracy, outperforming the shallow neural network’s 89.9% accuracy in the comparison analysis.
Zhou et al. [106]	Implemented visual analysis to detect money laundering and a multiview user interface to illustrate algorithm results and related transaction data.	Developed a visualization approach inspired by an abacus to analyze crypto transaction patterns, helping supervisors identify money laundering and understand the launderer’s trading tactics.	The algorithm’s performance is assessed using real-world data to demonstrate the effectiveness of the proposed method.
Liu et al. [107]	Introduced an improved graph embedding method for identifying money laundering in cryptocurrency.	Extracts real-time features to identify money laundering addresses by combining gas price and timestamps from transaction records to modulate random walk sampling, distinguishing involved nodes.	Validated with a custom dataset and tested with various classifiers, showing its effectiveness in extracting money laundering account features compared with other graph embedding techniques.
Yang et al. [108]	Proposed a two-tier framework for detecting crypto money laundering, combining heuristic rules and learning modalities.	Used statistical risk distribution features and transaction instances to create heuristic rules and combined LSTM and GCNN to identify patterns and used hard voting to strengthen the detection by fusing several anomaly detection algorithms.	Simplify acquiring labeled data for money laundering detection and enhancing the accuracy of unsupervised learning in recognizing money laundering activities.
Alarab and Prakoonwit [109]	Introduced a hybrid model that combines LSTM and GCN into a model called temporal GCN.	Applied active learning to a large Bitcoin transaction graph dataset. They used Monte Carlo-based adversarial attacks and dropout to handle uncertainties during active learning.	Compared their method with related works using the same dataset, finding that their method outperformed the others and evaluated two Bayesian methods against a baseline random sampling, yielding decent results.
Lo et al. [110]	Proposed a GNN-based framework with random forest classifier to detect money laundering activities.	Utilize a self-supervised graph isomorphism network (GIN), self-supervised deep graph infomax (DGI), and random forest classifier to detect money laundering in cryptocurrency.	Validated using the Elliptic dataset, yielding good results compared with the state-of-the-art across multiple performance metrics. It shows promise for identifying suspicious activities in crypto using self-supervised GNN.
Kolesnikova et al. [111]	proposed a stacked machine learning method to identify money laundering in virtual currencies.	Combines a random forest and graph convolutional network to identify potential shadow transactions, estimated to be around 2–3% of the market.	The emergence of crypto has led to new illicit transactions due to the anonymity provided by blockchain technology. This makes it difficult to track actual users, allowing attackers to conduct illicit transactions.
Yu et al. [112]	A deep learning-based approach was introduced to detect money laundering in cryptocurrency.	It includes an encoder to assess transaction significance based on flow amount values and utilizes a graph embedding method to address feature distortion. They used a graph autoencoder to capture transaction information, and the detector produced classification results using combined embedding.	The framework incorporates mutual learning with structure reconstruction and transaction classification losses, outperforming state-of-the-art techniques on the Elliptic dataset.
Wu et al. [113]	Introduced a multiclass dataset to address the Elliptic dataset limitations, which covers only some money laundering behaviors in the cryptoasset market.	Presented a framework to identify money laundering addresses originating from Ethereum theft incidents and examined cryptoattackers’ accounts. They used a taint analysis mechanism to track downstream transactions, identify service providers, and explore advanced money laundering techniques.	The experimental analysis revealed an increase in money laundering techniques, such as creating fake tokens and impersonating speculators.

**Table 7:** Comparison between the Sybil attack detection approaches.

Reference	Description	Technical Detail	Remarks
Swathi et al. [114]	Studied the influence of the Sybil attack on blockchain networks, particularly its impact on the system’s throughput.	They proposed a solution that enables nodes to monitor the behavior of other nodes and scrutinize those nodes that are transmitting blocks for a specific user.	These nodes are swiftly identified and added to a blocklist, with notifications sent to other nodes in the blockchain network to defend against Sybil attacks.
Geepthi et al. [115]	Introduced Sybil and Eclipse attack detection techniques based on fuzzy secure Kademlia (FSK).	The proposed method involves node authentication through an infrastructure server. Real-time network traffic is monitored for matching packet information.	The proposed framework has been proven effective in energy consumption and detection time.

## Data Availability

Not applicable.
